# Engineering performance and environmental assessment of sustainable concrete incorporating nano silica and metakaolin as cementitious materials

**DOI:** 10.1038/s41598-025-85358-8

**Published:** 2025-01-09

**Authors:** Namitha Raveendran, Vasugi Krishnan

**Affiliations:** 1 School of Civil Engineering, Vellore Institute of Technology, Chennai, Tamil Nadu 600127 India; 2 School of Civil Engineering, Vellore Institute of Technology, Chennai, Tamil Nadu 600127 India

**Keywords:** Durability, Eco strength efficiency, Embodied carbon, Mechanical behaviour, Metakaolin, Microstructural performance, Nano-silica, Engineering, Civil engineering, Nanoscale materials, Environmental impact

## Abstract

The carbon footprint associated with cement production, coupled with depletion of natural resources and climate change, underscores the need for sustainable alternatives. This study explores the effect of metakaolin (MK) and nano-silica (NS) on concrete’s engineering performance and environmental impact. Initially, compressive, tensile, and flexural strength tests, along with durability assessments like water absorption, sorptivity, rapid chloride permeability, and resistance to acid and sulphate attacks, were conducted. Later, X-ray Diffraction spectroscopy and Field-emission scanning electron microscopy were employed for microstructural analysis. Subsequently, the environmental impact of micro and nano materials was assessed using embodied carbon emissions and eco-strength efficiency. The results revealed that the hybrid mixes of 12.50% MK and 2% NS (M7) showed superior performance, demonstrating significant strength enhancements and eco-efficiency, achieving 0.15 MPa/kg CO_2_/m^3^ at 28th day. Meanwhile, the MK-only mix (M6) yielded the lowest embodied CO_2_ emissions at 330 kg CO_2_/m^3^. MK and NS effectively reduce porosity and enhance durability against environmental factors while lowering clinker content, contributing to sustainability. Furthermore, the microstructural behaviour showed early hydration, dense microstructure and additional Calcium Silicate Hydrate formation, leading to improved properties. The outcomes reveal that the concrete configuration has altered at micro and nano levels by the inclusion of MK and NS, demonstrating their substantial contribution to producing environmentally friendly, effective, and beneficial concrete.

## Introduction

### Background

Urbanisation and population growth have significantly increased resource consumption and environmental issues, making it crucial for the construction industry to develop sustainably^1^. Concrete, a widely used building material, substantially contributes to global CO_2_ emissions, energy consumption, and resource depletion. Consequently, its global consumption of 25 gigatons per year accounts for 10% of all man-made CO_2_ emissions^2,3^.

**Fig. 1:**
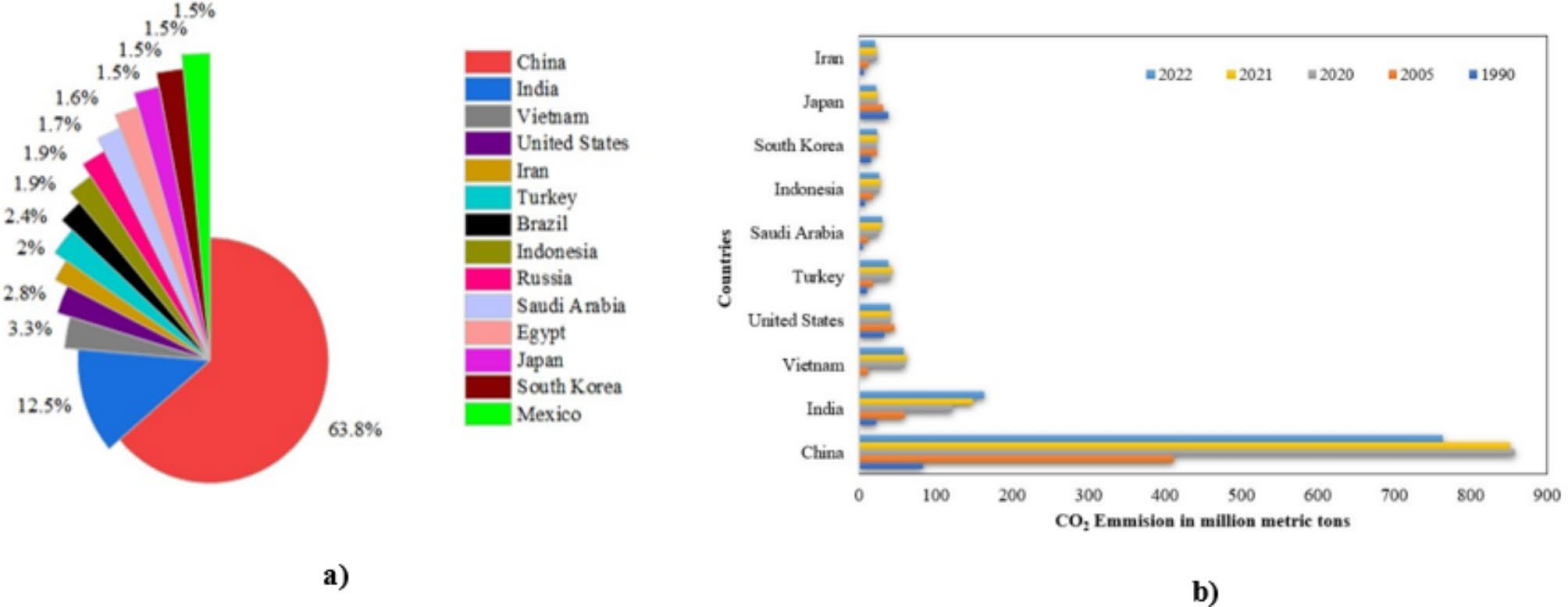
Global contribution of cement by various countries: (**a**) Cement production in 2023^21^ (**b**) carbon dioxide emissions^22^.

The worldwide cement output was estimated to reach 4 billion tons by 2023, with China and India holding the largest proportions, as seen in Fig. 1a. This sector accounts for 5–10% of worldwide anthropogenic CO_2_ emissions, with direct CO_2_ emissions per ton of cement increasing by 1.80% annually between 2015 and 2020. Figure 1b depicts the CO_2_ emissions from cement manufacturing in selected countries between 1990 and 2022. The International Energy Agency report for 2022 states that the cement sector is accountable for 7% of the worldwide CO_2_ emissions, with CO_2_ emissions per kilogram of cement made ranging from 0.66 to 0.82 kg^4,5^​. Regarding this, approximately 40% of the CO_2_ emissions are produced during the kiln process, with the remaining 50% produced during the roasting of limestone​. Moreover, the heat generated during the energy-intensive roasting process highlights the industry’s carbon-intensive and energy-consuming nature^6,7^. Therefore, minimizing the carbon footprint of the building industry will be critical in the fight against climate change. An essential tactic for achieving carbon neutrality is to lengthen the lives of buildings^8,9^. The work being described here intends to lessen the environmental damage caused by the manufacture of concrete and support international efforts to mitigate climate change^10^​. In order to accomplish Net Zero Emissions by 2050, a 3% yearly reduction in greenhouse gases is required until 2030^5^. For the implementation of Net Zero Emissions, the key strategies include improving fuel efficiency, reducing the clinker-to-cement ratio, using supplementary cementitious materials (SCMs), and creating alternative cement/binders without compromising cementitious properties^11^. One of the other effective methods is the use of geopolymers, which are inorganic polymers synthesized from industrial by-products like fly ash and slag, offering a sustainable and low-carbon alternative to traditional cement. Recent studies indicate that the binder type, such as slag and fly ash, significantly influences the mechanical properties and durability of geopolymers^12^. Additionally, factors like aggregate type and sulphate exposure impact the sorptivity, mechanical properties, and overall durability of geopolymers, making them a versatile and eco-friendly choice in sustainable construction^13–17^. Thus, for normal concrete, it is both practical and efficient to mitigate the environmental consequences of concrete manufacturing by employing secondary cementitious materials (SCM) including metakaolin (MK), mine waste, fly ash, ground granulated blast furnace slag and silica fume as alternatives to cement^18,19^. Among various SCMs, MK as a filler in cement has the potential to reduce CO_2_ emissions by over 170 kg per ton of cement^20^.

MK, derived from thermally treated kaolin (600–850 °C), enhances concrete’s mechanical properties and durability due to its increased pozzolanic activity. The primary characteristic of this pozzolanic transition product is the presence of highly active silica and alumina. This pozzolan reacts with the liberated Portlandite to generate more CSH gel^23^. Since Portlandite is a poor hydration product, it must be removed. Kostuch et al. state that in order to completely remove Portlandite, 20% cement substitution with MK is necessary^24^. Because of its pozzolanic properties and micro-pore filling, MK is attracting a lot of interest from researchers as a cement substitute^25^. Maximum strength development was achieved with 10% MK substitution, as demonstrated by Dinakar et al.^26^. The replacement of MK reduced durability attributes such as water permeability, chloride permeability, and water absorption. As a sustainable alternative to cement, MK improves concrete and reduces the carbon footprint. Research by Maddalena et al.^27^ shows that using 10% MK in concrete lowers CO_2_ emissions by 43%. Zeniesk et al.^19^ found that 10% MK replacement achieves the highest compressive strength and a 6% cost reduction per unit of strength. Unlike other SCMs, MK is manufactured to high standards, ensuring uniformity and high pozzolanic reactivity while reducing chloride migration and enhancing sulphate resistance, making it a promising material for sustainable, high-performance concrete^28,29^. Due to its pozzolanic and micropore-filling qualities, MK is being used increasingly as a cement substitute; nevertheless, it cannot fill nanopores in concrete, causing instability that compromises the material’s strength and longevity. Hence, over the past few decades, nanotechnology has enabled the manufacture of effective nanoparticles as an addition in mortar and concrete, enhancing their properties and lowering their environmental impact^30^.

Engineers and researchers are increasingly drawing on the application of nanoparticles, considered advanced, ultrafine or nanomaterials, in concrete technology. Diverse nanomaterials, including nano-alumina^31^, nano-silica (NS)^32^, nano-titanium oxide^33^, and nano-MK^34^ have been integrated into concrete to improve its fresh and hardened characteristics. Among various nanomaterials, NS significantly enhances the physical properties and strength of concrete^33^. It also contributes to the economic viability of concrete production by reducing the CO_2_ footprint^19^. Furthermore, substituting 2% of NS improves compressive strength over time by 15–21%^36^. Aside from the impact of NS on mechanical behavior, its effect on durability and long-term reliability of concrete is also extensively studied. Said et al. reported that adding 3% and 6% of NS to concrete reduced chloride ingression by 33% and 22%, respectively, and resulted in a low total charge during rapid chloride penetration tests, indicating the concrete’s high durability^35–37^. Moreover, NS at a dosage of 1 − 4%, also lowers total porosity from 24.50 to 32.10% which reduces the water absorption thus reflecting enhanced durability^38^.To develop better concrete performance, even dispersion of nanomaterials in the concrete mix is crucial. The dispersion and agglomeration of NS are primarily affected by particle size and dosage. Numerous studies have been undertaken to assess the influence of varying particle sizes and concentrations of NSa on the performance of concrete mixtures. Zhang et al. revealed that the incorporation of smaller NS particles (7 and 12 nm) at 2% of the cement’s weight resulted in enhanced cement strength and a more compact microstructure^36^. A study by Naji Givi et al. concluded that NS particles with a size of 15 nm might initially enhance concrete strength; however, cement would exhibit superior compressive strength after 90 days when 80 nm NS particles were utilized^39^. Furthermore, Hendrix et al. indicated that the ideal concentration of NS in a cementitious matrix for enhanced dispersion is 2%, with colloidal NS particles measuring 20 nm being the most effective among those sized between 5 and 75 nm^40^. To overcome such disparities, it is critical to optimize the optimal concentration of NS for all mix designs.

Utilizing micro or nanomaterials in concrete offers several advantages; however, traditional substitution methods have limitations in enhancing concrete properties. To increase the strength and performance of concrete technology, researchers are investigating hybrid fillers. The latest breakthrough in micro-nano blended hybrid concrete is that it enhances mechanical strength, freeze-thaw cycles, durability, and corrosion resistance. Furthermore, it is observed that the micro-nano effect regulates hydration, decreases shrinkage, and limits crack formation in the initial stages of curing^19,41,42^. Although various research projects, including the composite structures, have investigated diverse combinations of micro-nano materials, little has been done to explore concrete incorporating MK and NS^43,44^.

Furthermore, the environmental effects of MK and NS on concrete have also been under-researched. Thus, to address this gap, it is imperative to conduct studies regarding the notions of embodied energy and eco-strength efficiency. Embodied energy includes all the energy required to create a building material, while eco-strength efficiency evaluates the relationship between a material’s strength and its environmental impact^19,45^. Incorporating these concepts into building material design is crucial for minimizing greenhouse gas emissions and advancing both the longevity and ecological compatibility of contemporary infrastructure.

### Significance of research

Concrete containing SCMs is more ecologically friendly, has better technical qualities, and does not pose substantial global challenges. However, there is a notable lack of study on the influence of engineering and environmental impacts on micro-nano blended concrete. The novelty of this study is to explore the individual and combined effects of MK and NS in blended concrete, providing new insights into their synergistic influence while also evaluating their distinct impacts on engineering properties and environmental performance. Initially, the surface area, elemental composition, and phase analysis of the cement, MK, and NS were examined using Brunauer–Emmett–Teller (BET) analysis, X-ray fluorescence (XRF) and X-ray diffraction (XRD). Further, the morphology of each material was examined using FE-SEM. Subsequently, the mechanical properties, durability properties, and microstructural analysis were performed for various mixes, including conventional concrete (CC), lone MK, lone NS, and MK-NS blended concrete. Finally, the environmental impact of these concrete mixes was quantified in terms of Embodied energy and eco-strength efficiency. Ultimately, this research endeavours to provide valuable insights into the application of MK and NS in concrete to augment its strength, durability, and overall sustainability.

## Materials and methods

### Materials

In this study, Ordinary Portland Cement (OPC) of grade 53 was the cement used. It was acquired from Chettinad Cement Co. Ltd in India and fulfilled the requirements outlined in IS 12,269 − 2013, with an initial setting time of 80 min and a final setting time of 370 min, respectively. Herenba Instruments & Engineers, India, supplied MK and NS. These materials’ physical and chemical characteristics were evaluated using XRF spectroscopy and Indian Standard laboratory tests (IS 4031), as indicated in Table 1. The pH value and loss on ignition were calculated in accordance with the specifications provided in IS 877 (1989) and IS 1612:1976, respectively. The dispersion of NS and achievement of the desired slump value were facilitated by the use of Auramix-500, an altered polycarboxylate superplasticizer (SP). The coarse and fine aggregates obtained from a local supplier were subjected to testing according to the requirements specified in IS 2386-3. The coarse aggregates of 20 mm had a water absorption rate of 0.75 and a specific gravity of 2.70, whereas the fine aggregate, also known as M-sand, had a water absorption rate of 3% and a specific gravity of 2.66. The laboratory’s tap water at room temperature was utilized to mix the concrete.

**Table 1:** Assessment on characteristics of cementitious materials.

Physical properties										
Materials	Particle size	Specific gravity	Surface area (m^2^ /g)	pH	Photograph					
OPC	1–50 µm	3.15	1.07	12.50						
MK	0.60–1.40 µm	2.60	11	6						
NS	17 nm	2.30	202	4.15						

Chemical properties										
Materials	CaO	SiO_2_	Al_2_O_3_	SO_3_	Fe_2_O_3_	MgO	K_2_O	TiO_2_	Na_2_O	LOI
OPC	61.25	19.98	4.68	3.52	3.19	3.12	0.68	-	-	2.10
MK	0.09	52.00	46.00	-	0.60	0.03	0.03	0.65	0.10	0.50
NS	-	99.98	5e^−3^	-	1e^−3^	-	-	4e^−3^	-	0.66

*OPC ordinary portland cement; MK metakaolin; NS nano silica LOI Loss on ignition.

### Material characterization

Material characterisation is a structured investigation of the characteristics, phase compositions, and behaviour of a material. Materials science, engineering, and manufacturing are critical domains for ensuring the performance, durability, and dependability of materials based on their design and optimization. In this study, a comprehensive analysis of MK and NS was conducted using XRD and FE-SEM. XRD is a vital characterisation technique for determining the crystallite size and polymorph crystallinity of a material. Similarly, FE-SEM is used to find particle shape and microstructure of materials.

### Mix design

This study examined eight concrete mixes, highlighting the lone and hybrid effects of MK and NS. Table 2 presents the mix proportions for each concrete design, calculated according to the IS 10,262 − 2019 standard. Initially, NS was added as an additive material at various percentages of 1%, 2%, and 3% (M1-M3), while MK was used as a replacement material at percentages of 5%, 12.50%, and 20% (M4-M6). The control mix was designed with 100% OPC. Later, the hybrid effect of MK-NS blended concrete is examined (M7). Moreover, all mixes had 0.4 as water -binder ratio, with SP content varying from 0.20 − 0.60% of total cementitious content.

**Table 2:** Mix proportion of MK-NS modified concretes used in this study.

MIX ID	Cement (kg)	MK (kg)	NS (kg)	FA (kg)	CA (kg)	Water (kg)	SP (kg)
CC (MK-0%, NS-0%)	100	–	-	187.44	350.42	40	0.20
M1 (MK-0%, NS-1%)	100	–	1	187.44	350.42	40	0.30
M2 (MK-0%, NS-2%)	100	–	2	187.44	350.42	40	0.40
M3 (MK-0%, NS-3%)	100	–	3	187.44	350.42	40	0.50
M4 (MK-5%, NS-0%)	95	5	-	187.44	350.42	40	0.20
M5 (MK-12.50%, NS-0%)	87.50	12.50	-	187.44	350.42	40	0.30
M6 (MK-20%, NS-0%)	80	20	-	187.44	350.42	40	0.40
M7 (MK-12.50%, NS-2%)	87.50	12.50	2	187.44	350.42	40	0.60

*MK Metakaolin (replacement to cement); NS Nano silica (addition to cement); FA Fine aggregate; CA Coarse aggregate; SP Superplasticizer.

### Mixing and casting procedure

The concrete was blended in a pan mixer with a volume of 100 L. At first, aggregates were put in and blended for a duration of 2 min. Subsequently, cement, MK, and NS were introduced and mixed for an additional 2–3 min. Next, the dry mixture was combined with water and SP until a uniform consistency was obtained. To ensure the desired consistency, a slump test was performed before casting. The concrete was then poured into the appropriate moulds to test its mechanical and durability properties. Following a twenty-four-hour period at room temperature, the specimens were removed from the moulds and submerged in water for curing. Mechanical and durability tests were conducted on three specimens for each mix ratio combination on 7th, 28th, and 90th days.

### Testing procedure

#### Fresh properties

The slump cone test was conducted on all mixtures to ascertain consistency as well as workability of freshly mixed concrete. In this test, a frustum-shaped cone (slump cone) is placed on a smooth, non-porous surface and filling the cone with three layers of concrete and compacting them using a tamping rod. Upon filling the cone, it is meticulously elevated vertically, permitting the concrete to slump or settle to meet the workability standards as per IS 1199:1959^48^. The vertical distance from the apex of the cone to the highest point of the settled concrete is quantified in millimetres, reflecting the mix’s workability. A greater slump number indicates more workable concrete, whereas a lesser slump denotes a harder mixture. The test evaluates the ability of concrete to flow and fill moulds.

#### Mechanical properties

**Fig. 2:**
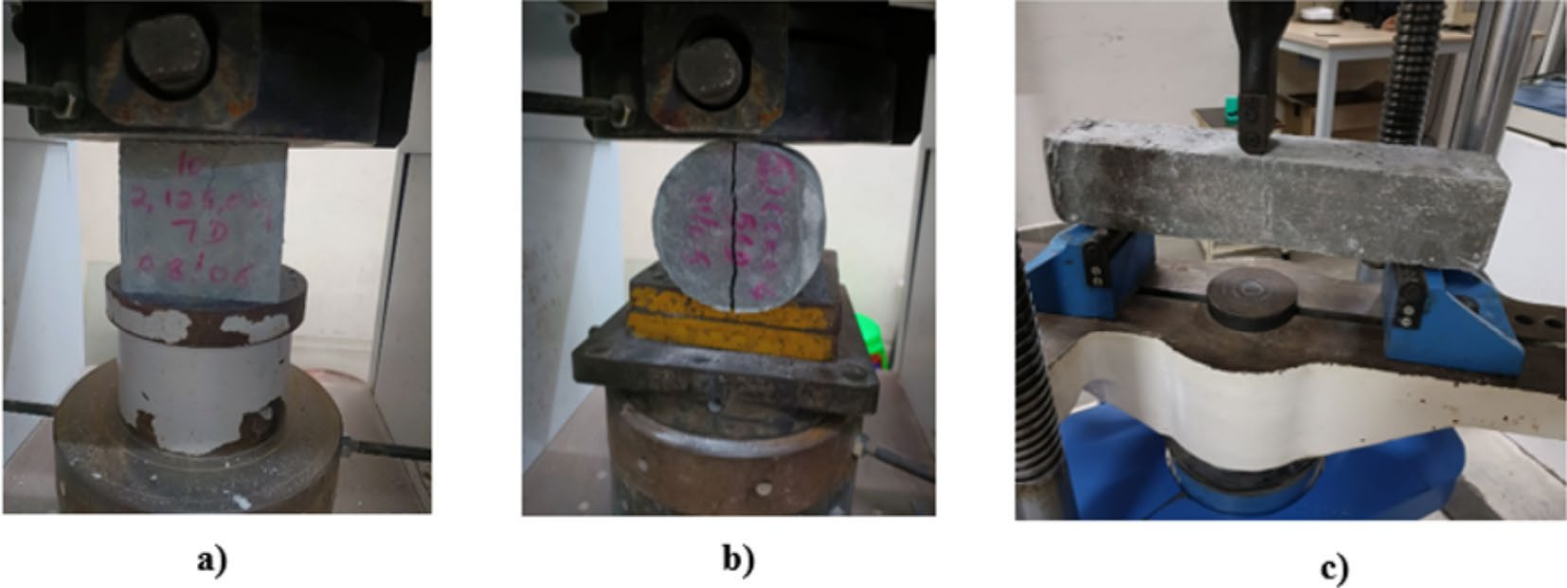
Test set up for mechanical properties (**a**) Compression (**b**) Splitting tensile (**c**) Flexural.

A 2000 kN capacity Compressive Testing Machine (CTM) was utilized for the compression and splitting tensile tests, while a 400 kN capacity Universal Testing Machine (UTM) was employed for the flexural strength test, in accordance with IS 516:1959^47^. Figure 2 illustrates the specimens’ experimental arrangement, including cube, cylinder, and prism samples specifically designed for compression, splitting, and flexural testing. The cube, cylinder and prism specimen were subjected to a loading rate of 0.60 MPa/sec, 0.05 MPa/sec, and 0.06 MPa/sec respectively, until it experienced ultimate fracture The analysis utilized the average results of three tests conducted on each sample to determine the ultimate strength, specifically in terms of compressive, splitting-tensile, and flexural properties.

#### Durability properties

### Water absorption

Water absorption test was performed to assess permeability characteristics. At first, the samples were oven-dried, cooled, weighed, and submerged in a curing tank until the weight stability was achieved. Later, the samples were weighed, and water absorption was calculated for the 28th and 90th days as per ASTM C642^50^ standard after being wiped with a dry absorbent cloth .

#### Sorptivity

**Fig. 3:**
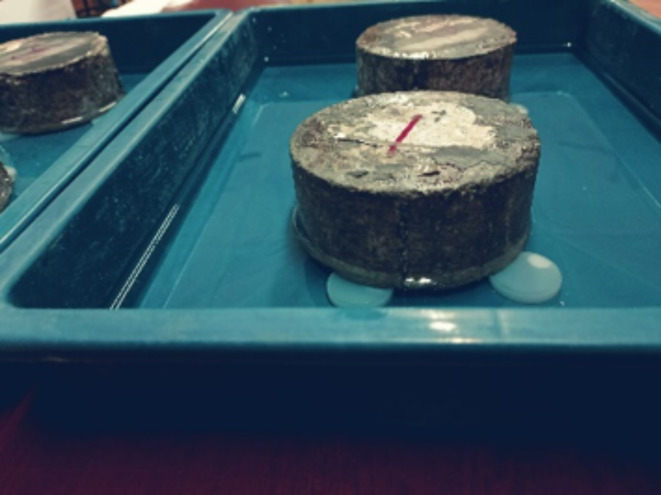
Test setup for sorptivity.

Sorptivity is a measure to access the amount of water absorbed by a concrete specimen through capillary action. Water was used as the test fluid, and the cylinders were submerged for 28th, and 90th days. The test specimen is a 100 mm diameter disc, with a length of 50 mm. Later, the side surfaces of the specimen were sealed using epoxy, and the non-exposed end was covered with a loosely attached plastic sheet secured with an elastic band. The specimen was conditioned in a desiccator inside an oven at 50 ± 2 °C for 3 days, as per ASTM C1585-13^51^. The relative humidity within the desiccator was controlled using a saturated solution of potassium bromide, ensuring that the specimen did not come into contact with the solution. After the conditioning period, the specimen was stored in a sealed container for a minimum of 15 days to allow for the equilibration of moisture distribution. This storage resulted in internal relative humidities of 50 to 70%. During the test, the water level in the pan was maintained 1 to 3 mm above the top of the support device, ensuring the exposed surface remained submerged. Furthermore, the specimen was placed in water up to 5 mm above the base after drying in an oven at 100 ± 10 °C, as shown in Fig. 3. ASTM C1585-13 was utilized to record test time variance and to compute sorptivity^49^.The process began with recording the initial mass of the specimen, followed by periodic mass measurements at specific intervals: 60 ± 2 s, 5 min ± 10 s, 10 min, 20 min, 30 min, and 60 min. Subsequent measurements were taken hourly up to 6 h. After the initial 6-hour period, measurements were taken once a day for 3 days, and additional measurements were recorded at 24-hour intervals for days 4 to 7. For days 7 to 9, one measurement was taken. This sequence ensured accurate tracking of water absorption over time.

#### RCPT

**Fig. 4:**
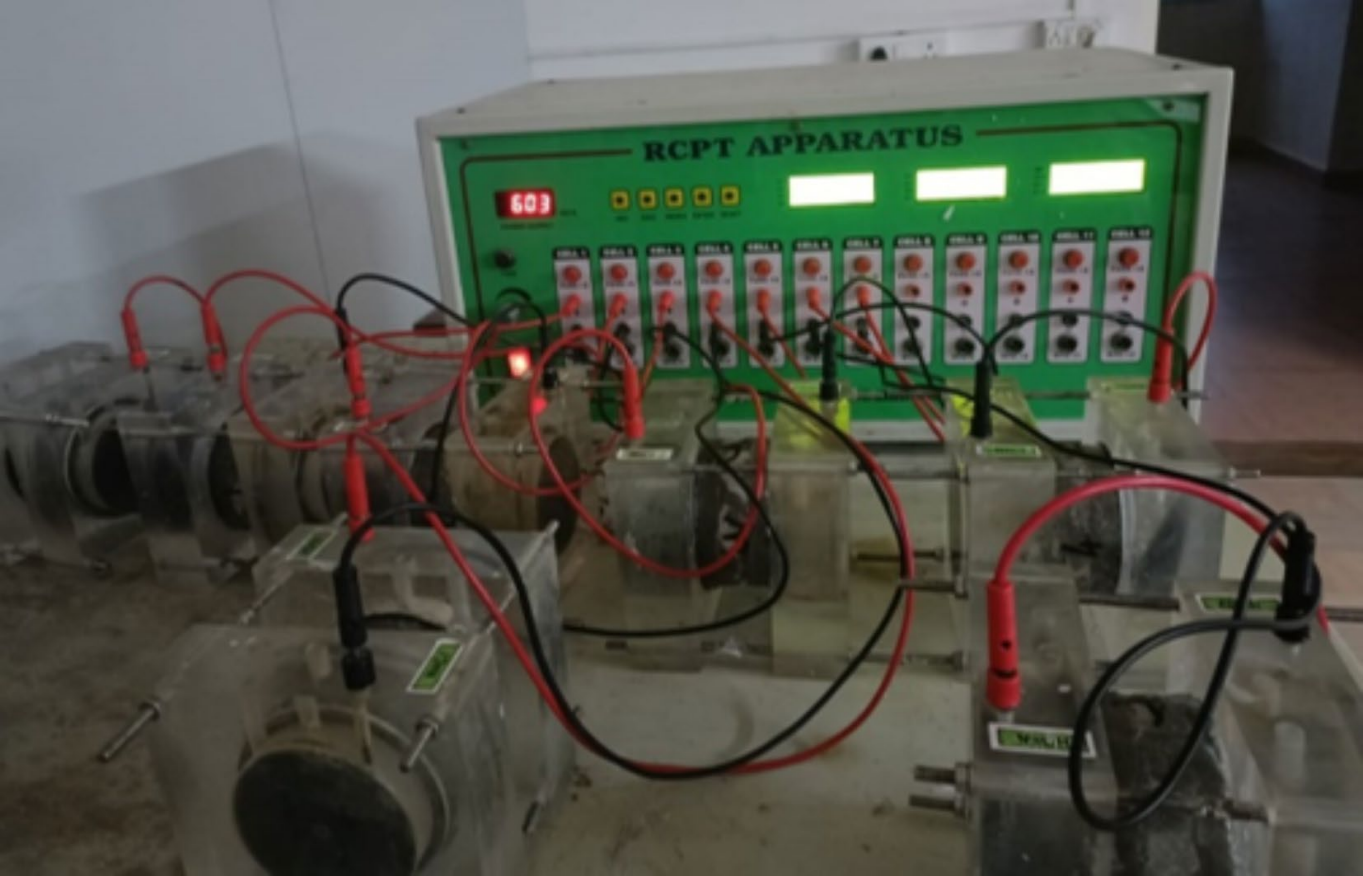
Test setup of the RCPT apparatus.

A rapid indication of concrete’s resistance against penetration of chloride ions is acquired by measuring the electrical conductance of concrete, is performed by RCPT test as per ASTM-C-1202 standard^50^. Initially, cylinders of 200 × 100 mm were casted, and then discs with a thickness of 50 mm and a diameter of 100 mm were cut from these cylinders. Within the RCPT cell, a solution containing 0.30 N sodium hydroxide is given to one side of the sample, while a solution containing 3% sodium chloride is administered to the opposite side of the sample. During the experiment, a constant voltage of 60 V DC was applied to the sample, and the current flowing through the sample was monitored at 30-minute intervals. Using Eq. (1), the total charge passed through the disc for six hours was measured for concrete quality evaluation as per ASTM-C-1202 ^52^. The test setup of the RCPT is illustrated in Fig. 4, where the test was conducted for 28th, and 90th days.

1 $$\:Q=900\left({I}_{0}+{2I}_{30}+{2I}_{60}+\cdots+{2I}_{300}+{2I}_{330}+{I}_{360}\right)$$

Where, Q is the charge passed (coulombs), I_o_ = current (amperes) immediately after the voltage is applied, and I_30_, I_60_, I_330_, and I_360_ represent the current in amperes passed after every 30 min.

#### Acid and sulphate resistance

A solution of 5% sulphuric acid as well as 5% sodium sulphate was made to evaluate the concrete’s resilience to acid and sulfate attacks. For the preparation of the acid solution, a 98% concentration of sulfuric acid was utilized. The pH of the acid solution was sustained below 7 by regularly refreshing the solution. A 28th day water cured concrete cube was immersed and cured for 90 days in sodium sulphate and sulphuric acid solutions. Later, using Eqs. (2) and (3), strength as well as weight loss were measured and compared to their initial values^51^.

2 $$\delta\:fc=\frac{\left(fc90-fc28\right)}{fc28}\times\:100$$

3 $$\delta\:w=\frac{\left(w90-w28\right)}{w28}\times\:100$$

Where, δfc = strength loss, δw = weight loss, fc28 = compressive strength at 28 days, w28 = weight at 28 days, fc90 = Residual strength at 90 days acid or sulphate curing, w90 = weight at 90 days acid or sulphate curing.

### Microstructural analysis

The microstructure of the concrete specimens was examined using XRD and FE-SEM. XRD, a method for identifying phase transitions in pozzolanic materials, was performed using a RIGAKU Smartlab 3 kW apparatus. Data were plotted at a 2θ angle between 5° and 80°. Phase identification is done using X’pert High Score software, and Origin-Pro was used to display the components. Additionally, the scattered electron method was employed in FE-SEM to ascertain the aggregation condition of micro and nano materials and the microstructure of the concrete samples. The mixes chosen for the FE-SEM investigation were CC, NS-MK blended concrete, and highest strength mixes of lone NS and lone MK.

### Environmental impact analysis

A systematic evaluation of the environmental impacts of concrete throughout its life cycle is included in an Environmental Impact Assessment (EIA). During this process, raw materials are extracted, manufactured, transported, constructed, and eventually disposed off. In addition to the environmental impacts of raw material extraction, energy consumption during production, transportation-related emissions, and potential disruptions during construction, there are also environmental concerns. It aims to identify adverse effects, mitigate them, and promote sustainable practices, such as energy efficiency, alternative materials, and responsible waste management. In general, the EIA of concrete contributes to sustainable development by helping make informed decisions to minimize its environmental footprint^19,52,53^. Therefore, lowering the CO_2_ footprint in the concrete sector is essential for reducing the effects of climate change and its related problems.

The amount of carbon embodied in concrete can be significantly reduced by partially substituting SCMs for clinker. As urbanization continues to increase, the need for durable, strong and sustainable concrete is rising. In light of this, concrete manufacturers are increasingly employing high-performance materials like MK and NS. Hence, the influence of SCM on total embodied CO_2_ emissions, and eco-strength efficiency was estimated in this study.

## Results and discussion

### Characterization of materials

**Fig. 5:**
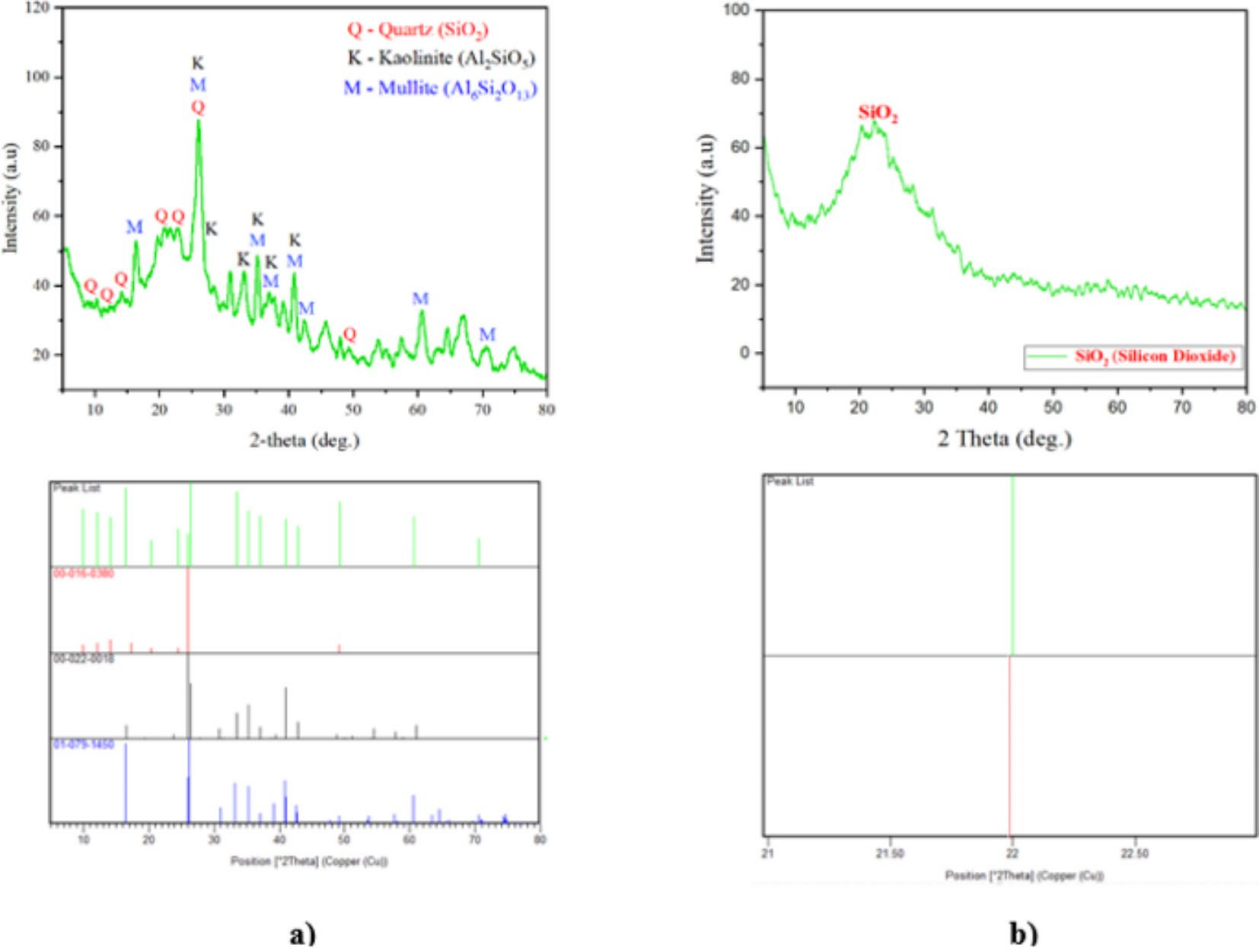
XRD analysis of SCMs (**a**) Metakaolin (**b**) Nano silica.

Within this study, the atomic composition, weight, shape, size and polymorph crystallinity of MK and NS were analysed using XRD and FE-SEM. Figure 5 represents the XRD pattern of MK and NS with their respective JCPDS card numbers. A large peak in the range of 15° to 35° was seen in the XRD spectrum of MK, as shown in Fig. 5a, suggesting a notable amount of amorphous powder. Quartz is identified as the major crystalline component, while kaolinite (K) and mullite (M). Similarly, the XRD analysis of NS is shown in Fig. 5b, where a notable 2θ peak at 21.90° is observed with an hkl value of (1 0 1), associated with the peak of amorphous SiO_2_. The XRD results highlight the potential of NS as a pozzolanic material due to its amorphous nature, which contributes to enhanced reactivity in cementitious systems.

**Fig. 6:**
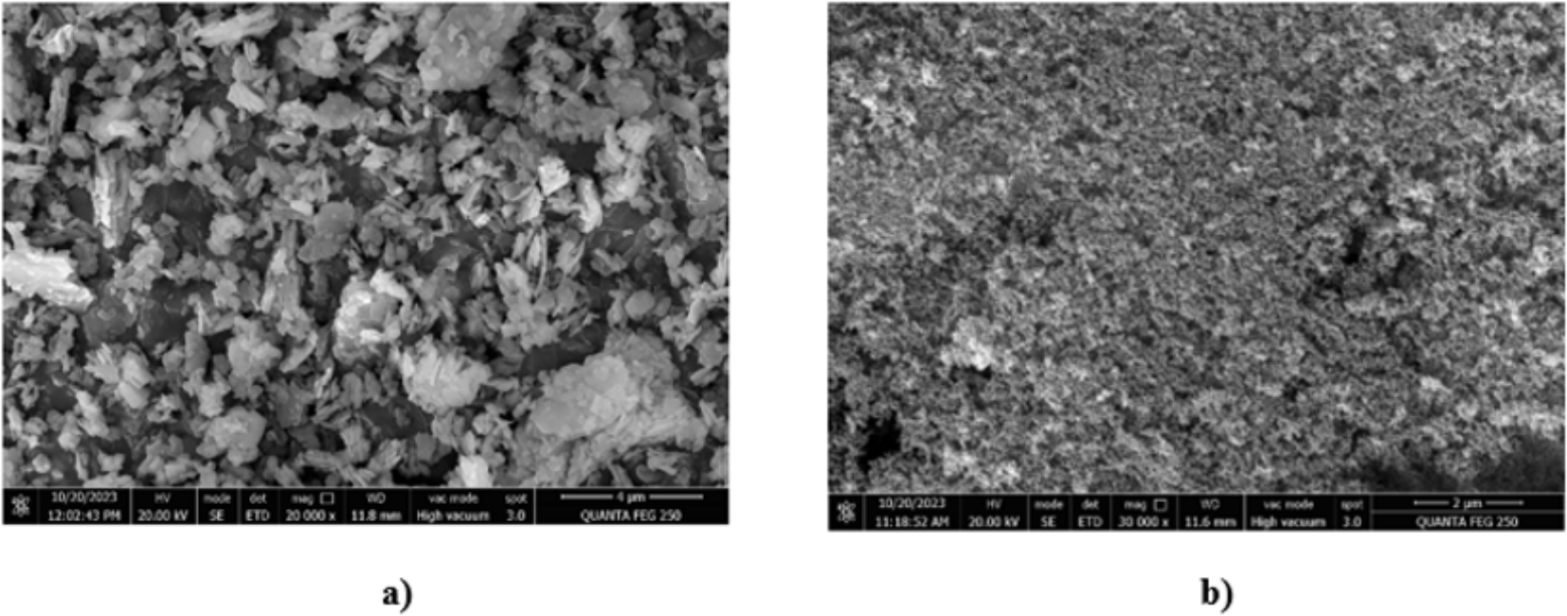
FE-SEM analysis of SCMs (**a**) Metakaolin (**b**) Nano silica.

The elemental composition and unique morphology of MK and NS were further examined using FE-SEM. The FE-SEM analysis of MK revealed a heterogeneous material with a dense matrix irregularly shaped and flake-like particles as depicted in Fig. 6a, which is in line with the results of other researchers^54,55^. The higher concentrations of silica and alumina found in MK have the capability to enhance the mechanical strength and longevity of concrete via the creation of calcium aluminate hydrates and calcium silicate. Additionally, snowflake-shaped aggregated particles exhibit sub-nanometer-range micropores, while microscopic silica crystals present apertures and voids between the particles, as shown in Fig. 6b. These morphological characteristics of NS indicate its ability to enhance the packing density and reduce the porosity of concrete, contributing to improved mechanical properties and durability.

### Fresh properties

To evaluate the workability of the various concrete mixes including MK and NS, a slump cone test was conducted. Following IS 456:2000, the dose of the superplasticizer (SP) was modified to maintain a slump of 75 ± 10 mm based on the amounts of MK and NS in each mix. Concrete mixes containing no more than 1% of the total cementitious material as admixture are needed to maintain a slump of 75 mm. Interestingly, concrete containing MK and NS reacts differently from conventional cement concrete because these inclusions have smaller particle sizes. The addition of NS at 1%, 2%, and 3% often reduces workability due to increased water demand and particle packing. Replacing cement with MK at 5%, 12.50%, and 20% also decreases workability due to its increased surface area and pozzolanic activity, which increases water demand. Accordingly, when the proportion of NS as an addition in the concrete varied from 1 to 3%, the SP proportion varied from 4 to 6%. In contrast, SP level was consistently maintained from 4 to 8% of total cementitious material when MK replacement at 5–20% was incorporated into the concrete. These variations demonstrate the impact of MK and NS on concrete workability and performance. By understanding the interaction between these materials and adjusting SP dosage accordingly, concrete mixes can be optimized for performance and sustainability. This approach highlights the potential for using MK and NS to develop more efficient, environmentally friendly concrete formulations.

### Mechanical properties of micro and nano concrete

**Fig. 7:**
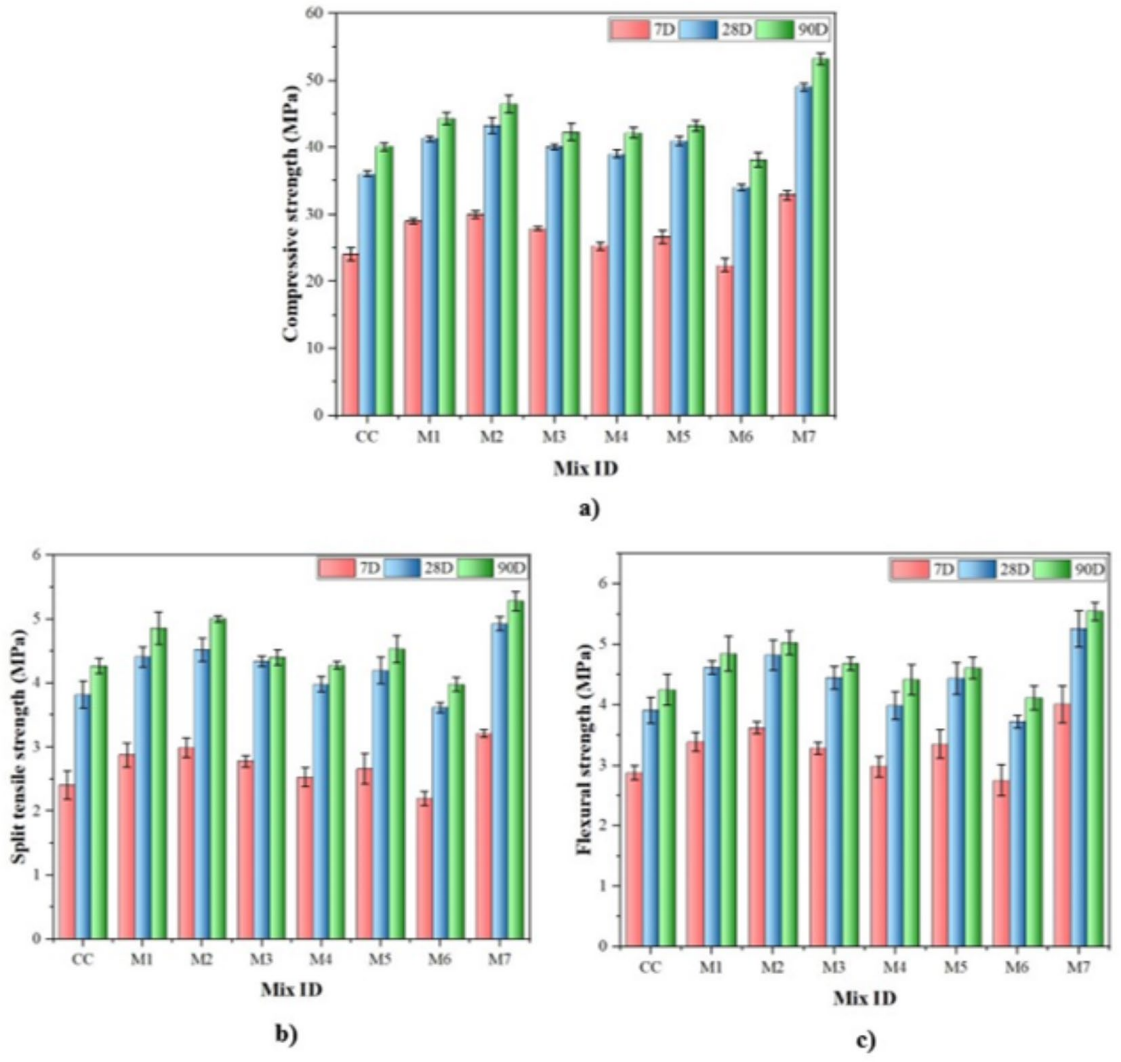
Effect of MK and NS on various mechanical properties (**a**) Compressive strength (**b**) Splitting tensile strength (**c**) Flexural strength.

Figure 7 illustrates MK and NS’s impact on concrete’s mechanical properties, specifically compressive, splitting tensile, and flexural strength. Initially, this study focused on the strength enhancement of numerous concrete mixes.

Mixes (M4-M6) with varying quantities of MK substitution (5-20%). The maximum enhancement in strength was seen at a concentration of 12.50%. The MK concentration is responsible for pore filling, increased cement hydration, and the pozzolanic reaction with Portlandite^23^. Specifically, at 7th, 28th, and 90th days, the compressive strength rose from 24.05 MPa to 26.66 MPa, 36.08 MPa to 41.02 MPa, and 40.16 MPa to 43.21 MPa compared to CC. Similarly, the splitting tensile strength shows an increment from 2.41 MPa to 2.66 MPa, 3.82 MPa to 4.20 MPa, and 4.27 MPa to 4.53 MPa, and flexural strength rises from 2.88 MPa to 3.35 MPa, 3.91 MPa to 4.44 MPa and 4.25 MPa to 4.61 MPa respectively. Although MK contributed to early-stage strength improvement, its impact became more significant as hydration progressed, enhancing microstructure development and overall strength. It played a more significant role in microstructure development, and its strength increased. However, increasing MK content to 20% reduced strength due to the dilution effect, due to the dilution effect, which offsets the benefits of pozzolanic activity^56^. Thus, as analysed from Figs. 7 and 12.50% MK is the optimal replacement level for achieving maximum mechanical strength due to optimal pozzolanic reaction and improved particle packing.

Later, the individual effects of NS on concrete were examined. It came to light that adding NS to OPC increased the concrete’s mechanical strength. However, the extent of improvement depended on the NS content, water-to-binder ratio, and curing time. NS has incorporated 1–3% proportions as an additive in concrete (M1-M3). In the presence of 2% NS, strength increased significantly, whereas, beyond 3%, all mechanical properties decreased because of NS particle agglomeration within the cement matrix. This occurs because NS particles, with their high surface area, demand more water for proper hydration and dispersion. Inadequate dispersion leads to unreacted particles accumulating and forming larger pores^57^. In this study, 2% NS was found to be the optimum addition percentage for concrete. Subsequently, mix 2, having 2% NS, shows improvement in strength at the 7th, 28th, and 90th days from 24.05 MPa to 30.02 MPa, 36.08 MPa to 43.28 MPa and 40.16 MPa to 46.52 MPa, respectively, which are 24.82%, 19.96% and 15.84% higher than CC. In line with compressive strength, the splitting tensile and flexural strengths displayed comparable increase patterns. Moreover, the strength increments of 24.07%, 18.32%, and 17.09% were noted for splitting tensile strength, whereas 24.39%, 22.64%, and 18.35% were observed for flexural strength when compared with CC. It is likely that the NS particles were highly refined, which increased the bond between the paste and aggregate, thereby increasing concrete strength^32^. In addition, the higher surface area of NS works as a filler material within the cement matrix, which enhances pozzolanic reactions and strength^58^. One of the important factors observed in this study was the early strength attainment of NS. This is because NS accelerates hydration due to the nucleation impact of smaller particles and enhanced packing of the mixes at the nanoscale. Additionally, NS exhibits pozzolanic reactivity, which reduces the amount of portlandite produced during hydration^39,59^.

It’s evident from the preceding discussion that adding NS at 2% and replacing MK at 12.50% are optimal dosages for concrete. The Mix M7 represented a hybrid combination of MK and NS at 12.50% and 2%, respectively. According to the compressive strength results, an increment in strength is seen from 24.05 MPa to 32.96 MPa, 36.08 MPa to 49.02 MPa, and 40.16 MPa to 53.21 MPa for 7th, 28th and 90th day. Likewise, splitting tensile strength also increased from 2.41 MPa to 3.21 MPa, 3.82 MPa to 4.93 MPa, and 4.27 MPa to 5.28 MPa for the 7th, 28th and 90th days. Similarly, flexural strength increased from 2.88 MPa to 4.01 MPa, 3.91 MPa to 5.26 MPa, and 4.25 MPa to 5.55 MPa on 7th, 28th and 90th days. Compared to CC concrete, strength increment percentages are approximately 30–40%, whereas for NS mixes, it is approximately 10–20%, and for MK mixes, it is approximately 20–30%. Based on the experimental results, it is evident that the hybrid effect had a greater effect on strength than the single effects of MK and NS. This is because the combined action of the MK and NS strengthens the pozzolanic reaction, increasing the concrete’s mechanical strength. Additionally, the concrete matrix is filled with nanosized silica, which strengthens and compacts the matrix. Similarly, the pozzolanic reaction of NS has a considerable impact on the interfacial transition zone (ITZ), leading to a decrease in crystal size and an increase in the formation of homogenous hydration products. This increases the binding capacity of aggregate sand cement paste, therefore enhancing the structural characteristics of concrete^60,61^.

### Comparison with previous research studies

In the study conducted by Shafiq et al. revealed that the 28th-day strength of NS addition concrete showed an increase of 5%, 7%, and 9% in compressive, splitting tensile, and flexural strength, respectively, compared to CC. For the lone MK mix, compressive, splitting tensile, and flexural strengths improved by 22%, 15%, and 16%, respectively. The optimized mixture exhibited the highest increases, with compressive, splitting tensile, and flexural strengths rising by 15.20%, 19.78%, and 32%, respectively, when compared to CC^62^. Meanwhile, Bhat. et al.^63^ found that the 28-day strength of concrete with NS addition demonstrated improvements of 11.22%, 10.71%, and 14.28% in compressive, splitting tensile, and flexural strength, respectively, compared to CC. For the concrete mixture containing only MK, the compressive, splitting tensile, and flexural strengths increased by 7.17%, 16.76%, and 9.64%, respectively. The optimized mixture, incorporating both MK and NS, showed the highest improvements, with compressive, splitting tensile, and flexural strengths increasing by 21.59%, 22.14%, and 16.77%, respectively, compared to CC. In the current study, the NS addition will give 10–20% improvement, MK replacement will give 20–30% improvement, and MK-NS blended concrete exhibits 30–40% increase for all mechanical properties when compared to CC, respectively, outperforming both previous experiments.

### Correlation between compressive strength and splitting tensile strength

**Fig. 8:**
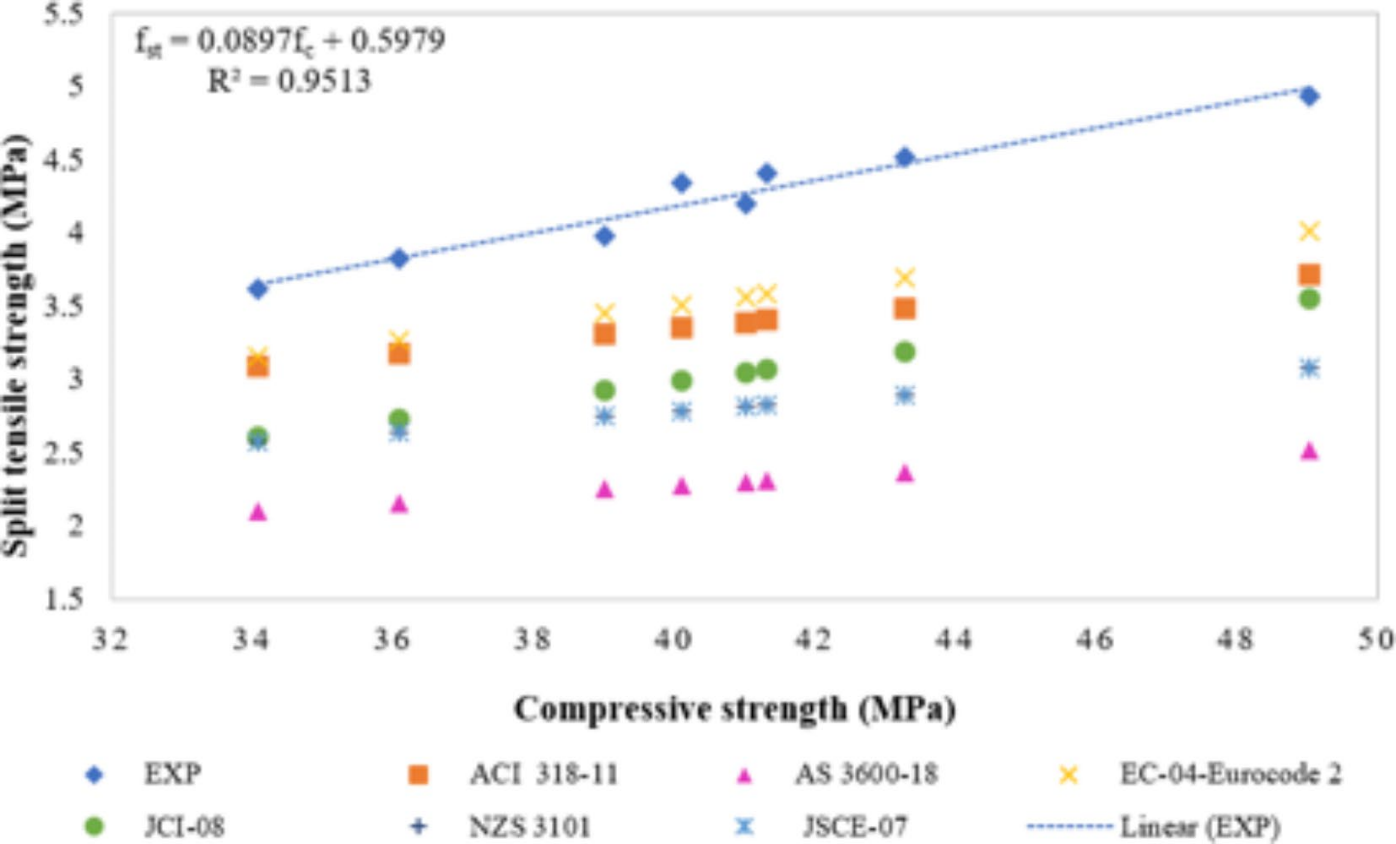
Correlation between compressive strength and splitting tensile strength.

The correlation between the compressive and splitting tensile strengths of the MK and NS-modified concrete are illustrated in Fig. 8. Various commonly used equations from different standards were employed to compare the results. These analytical computations can significantly reduce the number of laboratory tests needed for accurate strength estimation. The equations used in this study were based on the following standards: ACI 318 − 11 (Eq. 4), AS 3600-18 (Eq. 5), EC-04-Eurocode 2 (Eq. 6), JSCE-07(Eq. 7), JCI-08 (Eq. 8), and NZS 3101 (Eq. 9).

4 $$\:{f}_{St}=0.53\surd\:{f}_{c}$$

5 $$\:{f}_{St}=0.36\surd\:{f}_{c}$$

6 $$\:{f}_{St}=0.30({{f}_{c})}^{\frac{2}{3}}$$

7 $$\:{f}_{St}=0.44\surd\:{f}_{c}$$

8 $$\:{f}_{St}=0.13({{f}_{c})}^{0.85}$$

9 $$\:{f}_{St}=0.44\surd\:{f}_{c}$$

where fst is splitting tensile strength in (MPa), and fc is the compressive strength in (MPa).

Figure 8 indicates that the observed splitting tensile strengths in the experiment were much greater than the predicted values obtained from all standards. Out of the several standards available, EC-04-Eurocode 2 provides the most precise forecasts and is highly consistent with actual experimental results.

Equation 10 shows a significant linear connection between the splitting tensile and compressive strengths, indicating a high degree of correlation. This is corroborated by the high coefficient of determination of 0.95, that confirms the reliability of the derived equation.

10 $$\:{f}_{St}=0.0897\:{f}_{c}+ 0.5979$$

### Correlation between compressive strength and flexural strength

**Fig. 9:**
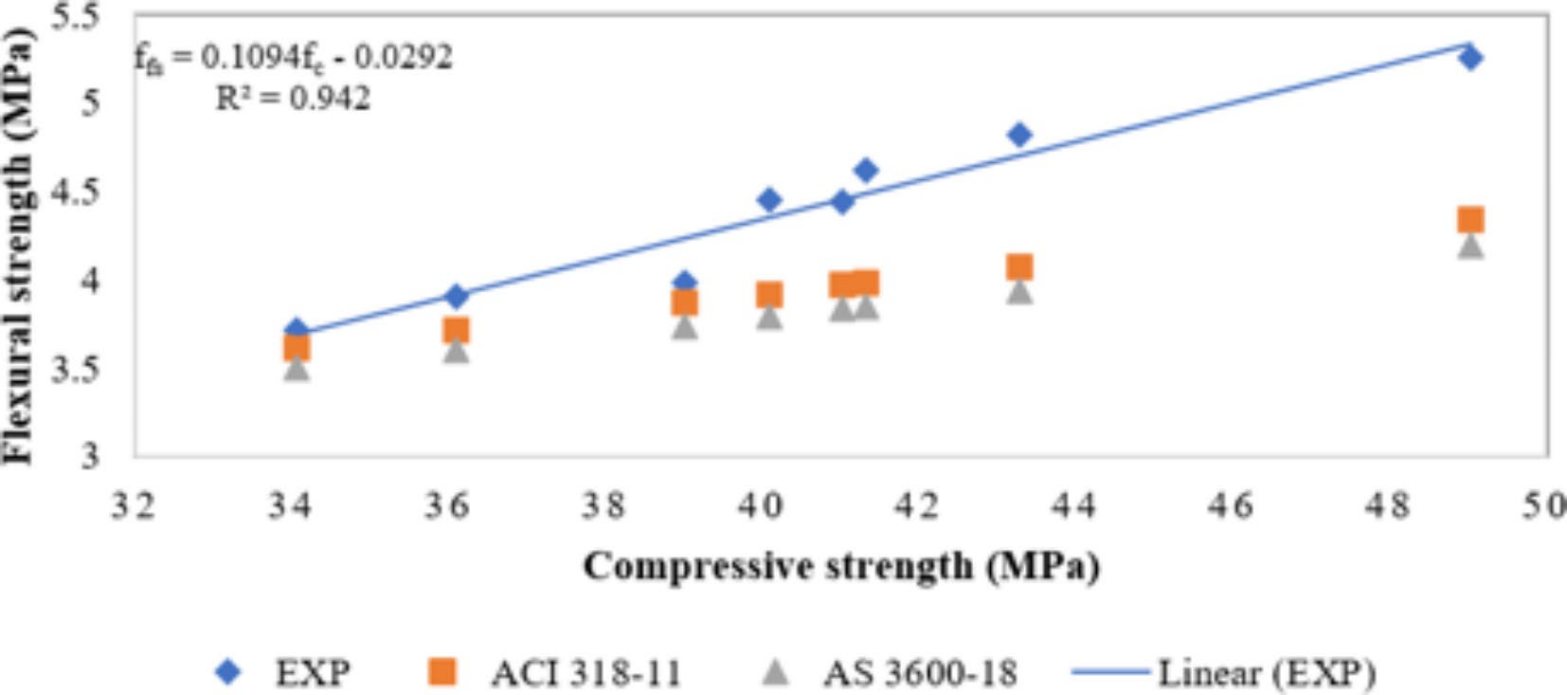
Correlation between compressive strength and flexural strength.

Figure 9 illustrates the correlation between the compressive strength and flexural strengths of NS-MK-modified concrete. This correlation was established using experimental data and computations of various standards conducted over a period of 28 days. Multiple equations were used to forecast flexural strength using the data of compressive strength data done experimentally, utilizing the ACI 318 − 11 (Eq. 11) and AS 3600-18 (Eq. 12) standards, as follows:

11 $$\:{f}_{fs}=0.62\surd\:{f}_{c}$$

12 $$\:{f}_{fs}=0.60\surd\:{f}_{c}$$

where f_fs_ is the flexural strength in (MPa) and f_c_ is the compressive strength in (MPa).

The graph showed that the experimental flexural strengths were significantly higher than the anticipated values derived from all established benchmarks. The experimental results also demonstrated a high level of reliability, as indicated by the R^2^ value of 0.94 at 28th day. The linear equation representing this relationship is as follows:

13 $$\:{f}_{fs}=0.1094\:{f}_{c}-0.0292 $$

### Durability properties

#### Water absorption

**Fig. 10:**
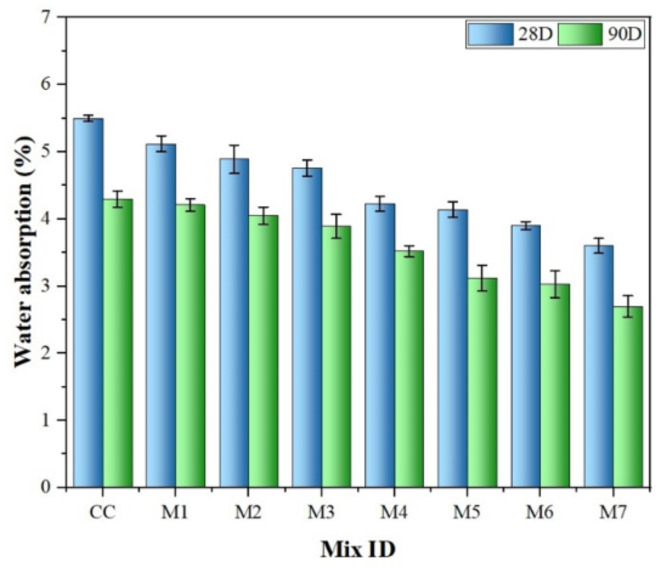
Effect of MK and NS on water absorption at 28th and 90th days.

Water absorption testing via total immersion is a crucial measure of concrete durability, indicating potential issues with faster and deeper water penetration. Factors such as porosity, density, and curing conditions significantly influence water absorption. Concrete mixes containing additives, such as MK and NS, show a widening gap in water absorption levels with higher replacement ratios at 28th and 90th days, as shown in Fig. 10. The results typically range from 3.60 to 4.90% at 28th day, indicating good concrete quality. The study found that increasing the content of NS in concrete led to a decrease in water absorption, as nanopores were filled due to nano particles of NS particles, thereby reducing the depth of water penetration^60^. Furthermore, the incorporation of MK results in decreased water absorption owing to pore filling and pozzolanic reactivity^29,64^. The most significant reduction occurred with a 20% MK content, with further enhancements observed when MK and NS were combined. Mixtures containing MK-20% and NS-2% demonstrated better performance, enhancing paste bonding characteristics and pore structure, thus enhancing overall concrete durability^62,63^.

### Sorptivity

**Fig. 11:**
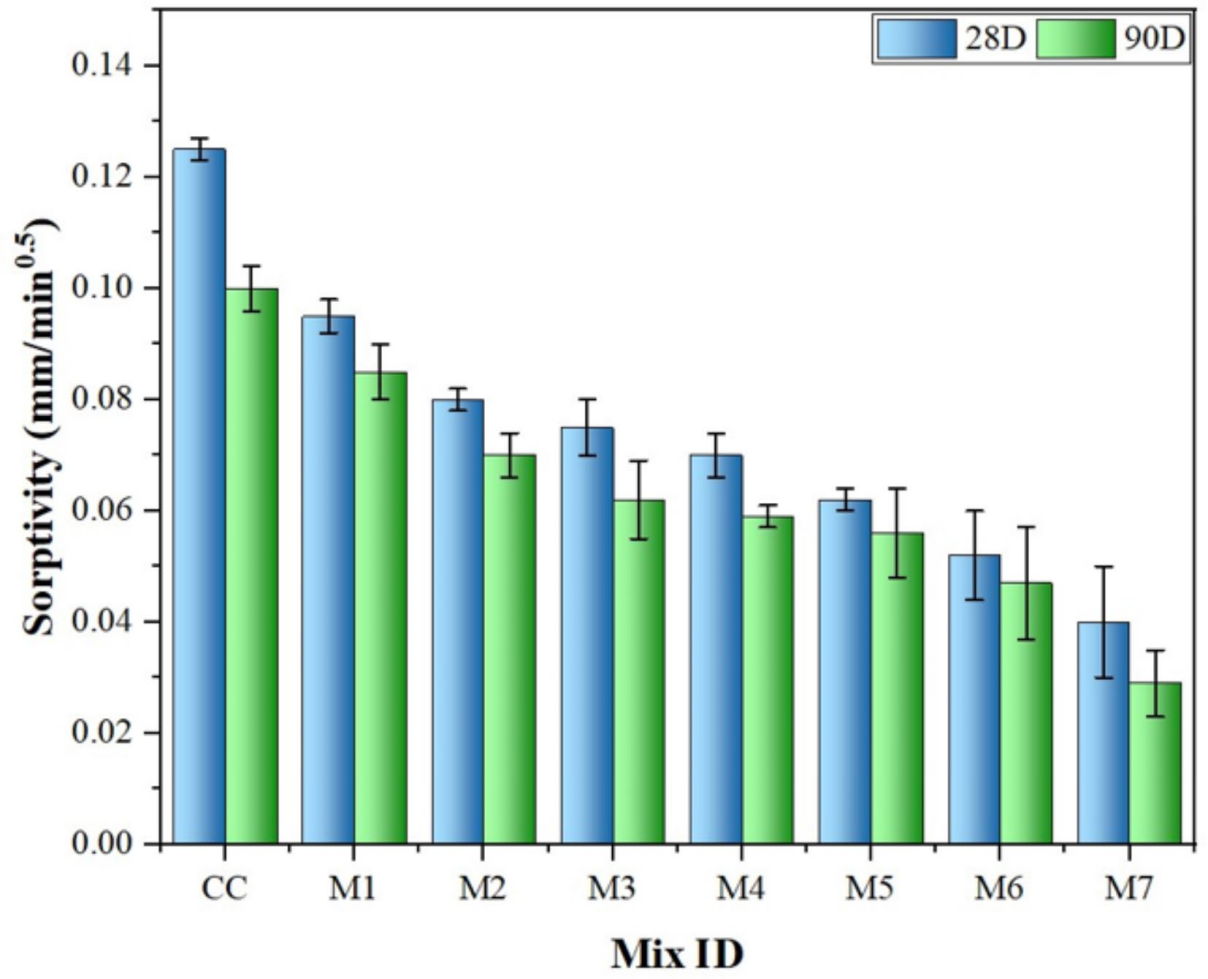
Effect of MK and NS on sorptivity at 28th and 90th days.

Sorptivity is a method for measuring the rate at which water is drawn into concrete pores by capillary action. As illustrated in Fig. 11, all concretes were tested for sorptivity at different curing ages. It is evident from the Fig. 11 that the sorptivity values for concrete containing MK and NS at 28th and 90th days are considerably lower than those for the control concrete. The study demonstrated that the MK-modified concrete (M4-M6 mixes) performed better than the CC, significantly improving water penetration through capillary suction. This improvement is due to the interaction between MK and calcium hydroxide, which creates additional cementitious products and enhances the microstructure of the concrete. This process reduces pore connectivity and water movement, thereby decreasing sorptivity^28,65^. Furthermore, the high pozzolanic reactivity of NS also contributes to the formation of more hydration products, filling capillary pores and reducing their connectivity, which limits water ingress pathways (M1-M3 mixes). This results in a denser microstructure and decreased water sorptivity^58^. Additionally, the most significant decrease in sorptivity is shown by MK-NS-modified concrete (M7), which enhances the packing density of concrete particles. This leads to a more compact microstructure and further reduces the water sorptivity.

### Rapid chloride permeability test

**Fig. 12:**
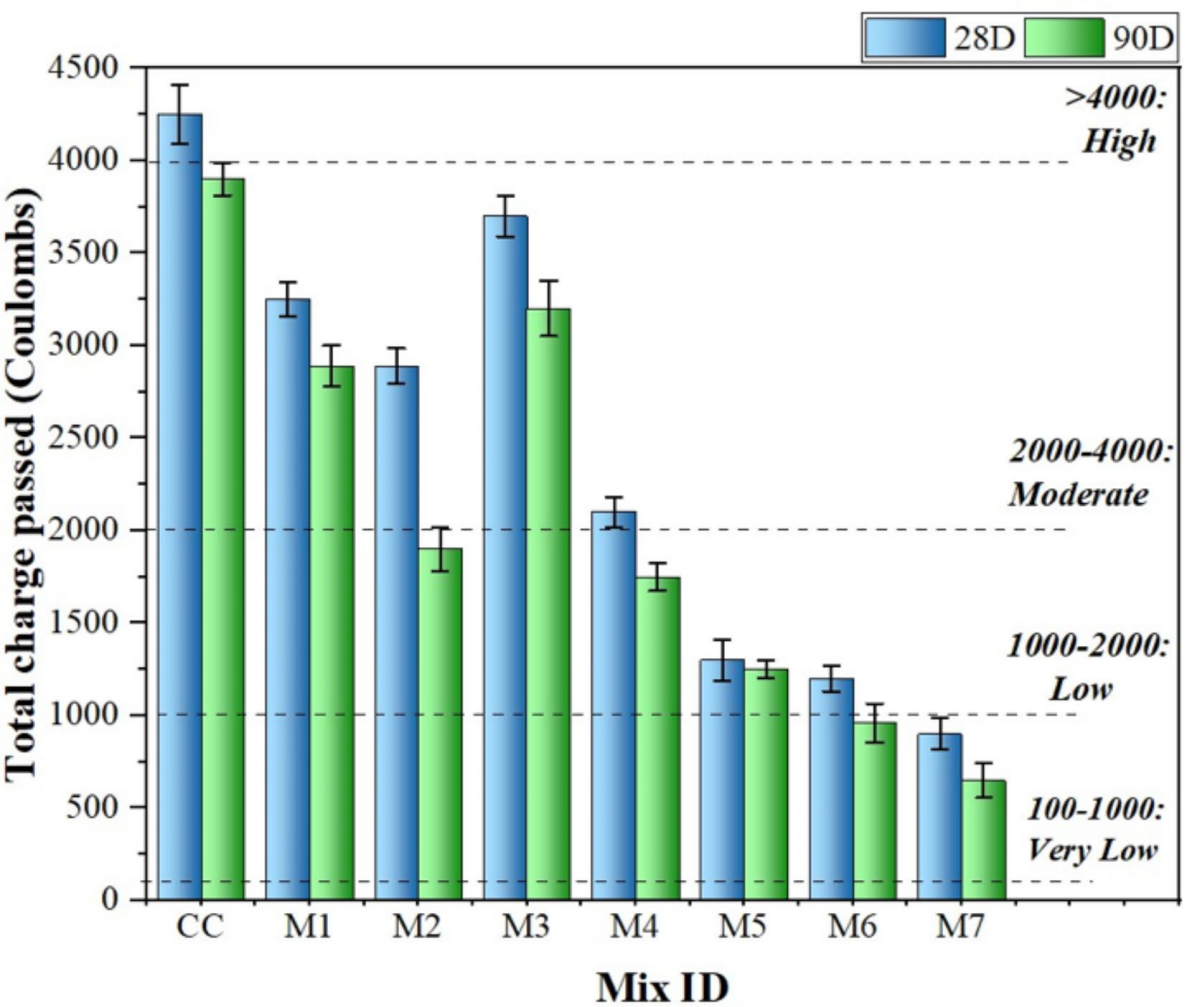
Effect of various mixes on RCPT at 28th and 90th days.

It is vital for concrete structures to be able to resist the intrusion of chloride ions. In this study, all mixes were subjected to an accelerated chloride permeability test at 28th and 90th days, and the results are shown in Fig. 12, along with the total charge passing in six hours with the aid of ASTM C1202.

The investigation revealed that all MK mixes (M4-M6) had very low to moderate chloride permeabilities in the 980–2100 C range. Moreover, the CC mix had a high chloride permeability of approximately 4230 C, indicating that the MK-containing mixtures performed substantially better^66^. The MK concentration led to a dramatic reduction in the barrier to chloride ion penetration. Adding MK reduces steel reinforcement corrosion because the system is better at binding chloride ions, reducing the free chlorine ions available to cause corrosion. By reacting with chloride ions in hydrated cement, C_3_A forms inert products, such as Friedel’s salt, which helps in chemically binding chloride ions, thereby improving durability^67,68^. Furthermore, all the NS mixes (M1-M3) had low to moderate chloride permeabilities. In contrast to the CC mix, NS, with its nano-pore-filling properties and pozzolanic reactions, densifies concrete and lowers free chloride ion content, enhancing the resistance against chloride penetration^69^. Additionally, agglomeration and large pores caused by 3% NS (M3 mix) may result in the ingression of chloride ions deeper into concrete mixes, rising RCPT values^70^.

Furthermore, a low charge passed (Coulombs) value is also observed in Fig. 12 for the MK-NS blended concrete (mix 7). According to ASTM C1202, any concrete mix with an RCPT value of less than 1000 C will have long-term durability throughout the life of the structure. The enhanced pore structure by NS and the high aluminate concentration in MK are the leading causes of this improvement. In the case of MK, the chlorides undergo a reaction with the aluminates to produce Fredel’s salt, enabling the chemical bonding of chloride ions with aluminates. As a result, their capacity to infiltrate the concrete matrix is reduced^59,71^.

### Acid attack

#### Acid mass loss and strength loss factors

**Fig. 13:**
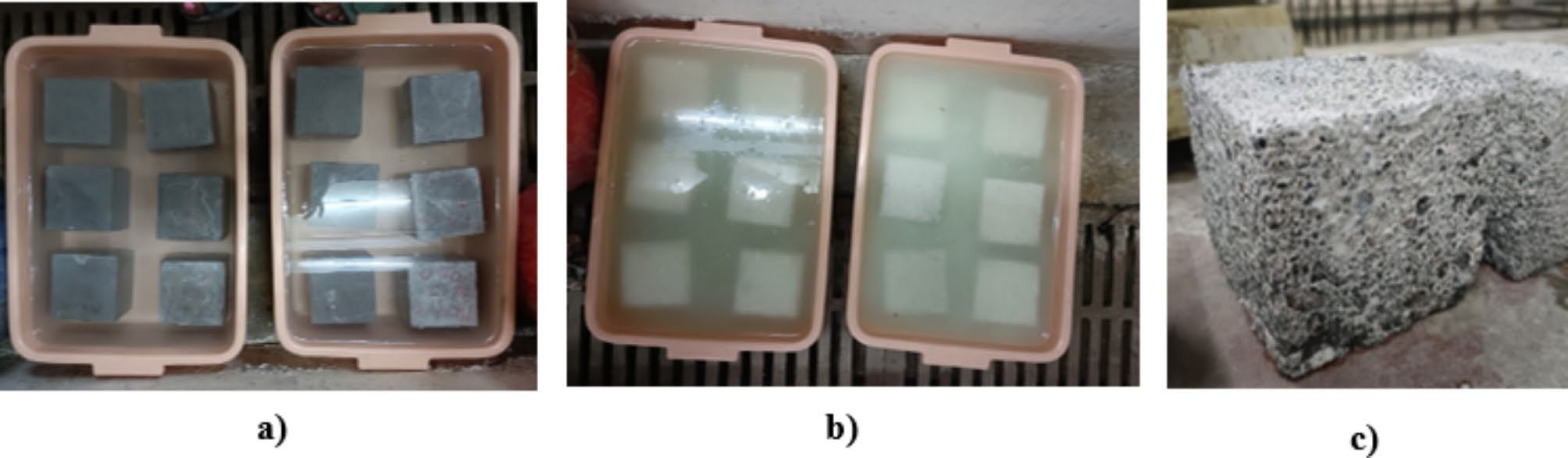
Effect of acid attack (**a**) before acid curing (**b**) after acid curing (**c**) white matter deposited on surface of specimen.

**Fig. 14:**
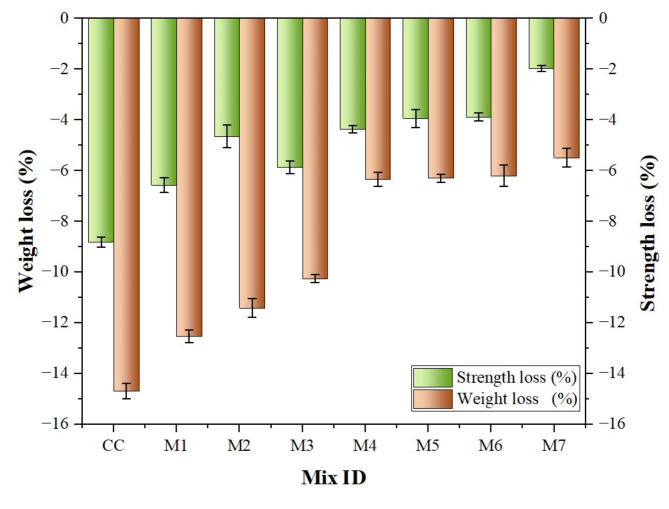
Effect of weight loss and strength loss after acid attack at 90th day.

The visual appearance of the specimens before and after the acid attack is illustrated in Fig. 13. The mass and strength data of the specimens before and after being immersed in acidic solutions for 7th, 28th and 90th days were conducted, as shown in Table 3. Notably, the greatest loss in strength and weight occurred after 90th day. Consequently, a detailed analysis was conducted to determine the specific percentage of mass loss and strength loss at 90th day, as shown in Fig. 14.

As mentioned above, the investigation revealed that after exposure to sulphuric acid, all the concrete specimens experienced reductions in both weight and strength. The maximum mass reduction observed in the case of CC was 8.80%. However, modified concrete specimens incorporating MK and NS showed some level of resistance. The MK-NS blended concrete had a minimal mass loss of approximately 2%. This is because the extended exposure of concrete to an acidic environment can lead to acid attack, where sulphate ions dissolve calcium silicate (C-S) layer as well as degrade C-H into a white substance. Further, these white matter accumulates on the specimen’s surface, wears off, and exposes a new layer to the acidic medium, which causes mass loss^63^. Likewise, the compressive strength declined for all specimens, with a minimum loss of 5.48% for MK-NS blended concrete and 14.69% for the CC. In acidic environments, calcium sulphate and ettringite are formed when sulphuric acid reacts with hydration products, resulting in volume changes and concrete degradation. Furthermore, the deterioration rates of the blended cement concrete specimens were lower than those of the control mix, owing to less disintegration.

**Table 3:** Effect of weight loss and strength loss after acid attack at 7th, 28th and 90th days.

Mix ID	Initial compressive strength	Strength after acid exposure (MPa)			Original weight	Weight of the specimen after acid attack (kg)		
	0D	7D	28D	90D	0D	7D	28D	90D
CC	26.96	24.26	23.92	23.00	2.61	2.50	2.42	2.38
M1	28.02	27.63	26.21	24.51	2.59	2.54	2.52	2.42
M2	29.18	28.12	27.79	25.85	2.58	2.51	2.47	2.46
M3	27.21	27.02	26.21	24.42	2.56	2.49	2.43	2.41
M4	26.03	25.69	24.23	23.71	2.52	2.42	2.38	2.36
M5	22.85	22.13	21.69	20.98	2.54	2.45	2.40	2.38
M6	26.85	26.01	25.52	24.99	2.58	2.55	2.50	2.42
M7	33.92	33.11	32.52	32.06	2.53	2.50	2.49	2.48

### Acid durability and attack factor

The acid durability factor (ADF) of concrete indicates its capacity to withstand acid attack without major damage. It is influenced by variables such as the acid type and concentration, concrete mix composition, and curing conditions. It can be assessed using methods that measure the mass loss or penetration depth of the exposed concrete samples^57^. Higher values indicated a higher level of acid corrosion resistance. The Acid Attack Factor (AAF) measures the extent of corrosion at the corners of a cube, determined by measuring the diagonals between the affected face and the opposite faces^72^. Here, a higher value signifies greater damage. In acid-sensitive areas, knowledge of this component is essential for guaranteeing the longevity and resilience of concrete structures. ADF and AAF can be calculated using Eq. (14) and Eq. (15).

14 $$\:ADF={S}_{r}\left(\frac{N}{M}\right)$$

15 $$\:\text{A}\text{A}\text{F}=\frac{\text{L}\text{o}\text{s}\text{s}\:\text{i}\text{n}\:\text{m}\text{m}\:\text{o}\text{n}\:\text{e}\text{i}\text{g}\text{h}\text{t}\:\text{c}\text{o}\text{r}\text{n}\text{e}\text{r}\text{s}\:\text{o}\text{f}\:2\:\text{c}\text{u}\text{b}\text{e}\text{s}}{4}$$

Where S_r_ is the relative strength.

S_r_ = Relative strength.

N = Duration of immersion or termination period.

M = Duration of the test.

16 $$\:\text{R}\text{e}\text{l}\text{a}\text{t}\text{i}\text{v}\text{e}\:\text{s}\text{t}\text{r}\text{e}\text{n}\text{g}\text{t}\text{h}\:=\frac{\text{S}\text{t}\text{r}\text{e}\text{n}\text{g}\text{t}\text{h}\:\text{a}\text{f}\text{t}\text{e}\text{r}\:\text{a}\text{c}\text{i}\text{d}\:\text{e}\text{x}\text{p}\text{o}\text{s}\text{u}\text{r}\text{e}\:}{\text{I}\text{n}\text{i}\text{t}\text{i}\text{a}\text{l}\:\text{c}\text{o}\text{m}\text{p}\text{r}\text{e}\text{s}\text{s}\text{i}\text{v}\text{e}\:\text{s}\text{t}\text{r}\text{e}\text{n}\text{g}\text{t}\text{h}}$$

**Table 4:** Evaluation of the ADF of specimens exposed to acidic solution.

Mix ID	Initial compressive strength	Strength after acid exposure (MPa)			Relative strength (S_*r*_)			ADF			AAF		
		7D	28D	90D	7D	28D	90D	7D	28D	90D	7D	28D	90D
CC	26.96	24.26	23.92	23.00	0.90	0.89	0.85	0.07	0.29	0.85	0.40	0.75	1.40
M1	28.02	27.63	26.21	24.51	0.99	0.94	0.87	0.08	0.29	0.87	0.38	0.63	1.31
M2	29.18	28.12	27.79	25.85	0.96	0.95	0.89	0.07	0.30	0.89	0.32	0.52	1.25
M3	27.21	27.02	26.21	24.42	0.99	0.96	0.90	0.08	0.30	0.90	0.29	0.42	1.01
M4	26.03	25.69	24.23	23.71	0.99	0.93	0.91	0.08	0.29	0.91	0.28	0.43	0.95
M5	22.85	22.13	21.69	20.98	0.97	0.95	0.92	0.08	0.30	0.92	0.21	0.39	0.83
M6	26.85	26.01	25.52	24.99	0.97	0.95	0.93	0.08	0.30	0.93	0.19	0.32	0.76
M7	33.92	33.11	32.52	32.06	0.98	0.96	0.95	0.08	0.30	0.95	0.13	0.25	0.55

The ADF and AAF were determined at 7th, 28th, and 90th days from the relative strength, as shown in Table 4, and the results showed that the mix 7 is more resistant. NS has high surface area and is more reactive, which enhances pozzolanic activity when mixed with cementitious materials such as MK^57^. As a result, a C-S-H gel is produced, which increases concrete’s densification and decreases its susceptibility to acid attacks. Meanwhile, MK enhances the production of extra C-S-H gel and decreases the amount of calcium hydroxide in the concrete matrix, resulting in reduced acid resistance^68^. Moreover, the combined impact of MK and NS enhances the quality of the pore structure, decreases the connectivity of pores, and strengthens the cement matrix, resulting in a reduction of paths for acid intrusion^63^. This results in a higher ADF and lower AAF in the MK-NS blended concrete when compared to all other mixes.

### Sulphate attack

**Fig. 15:**
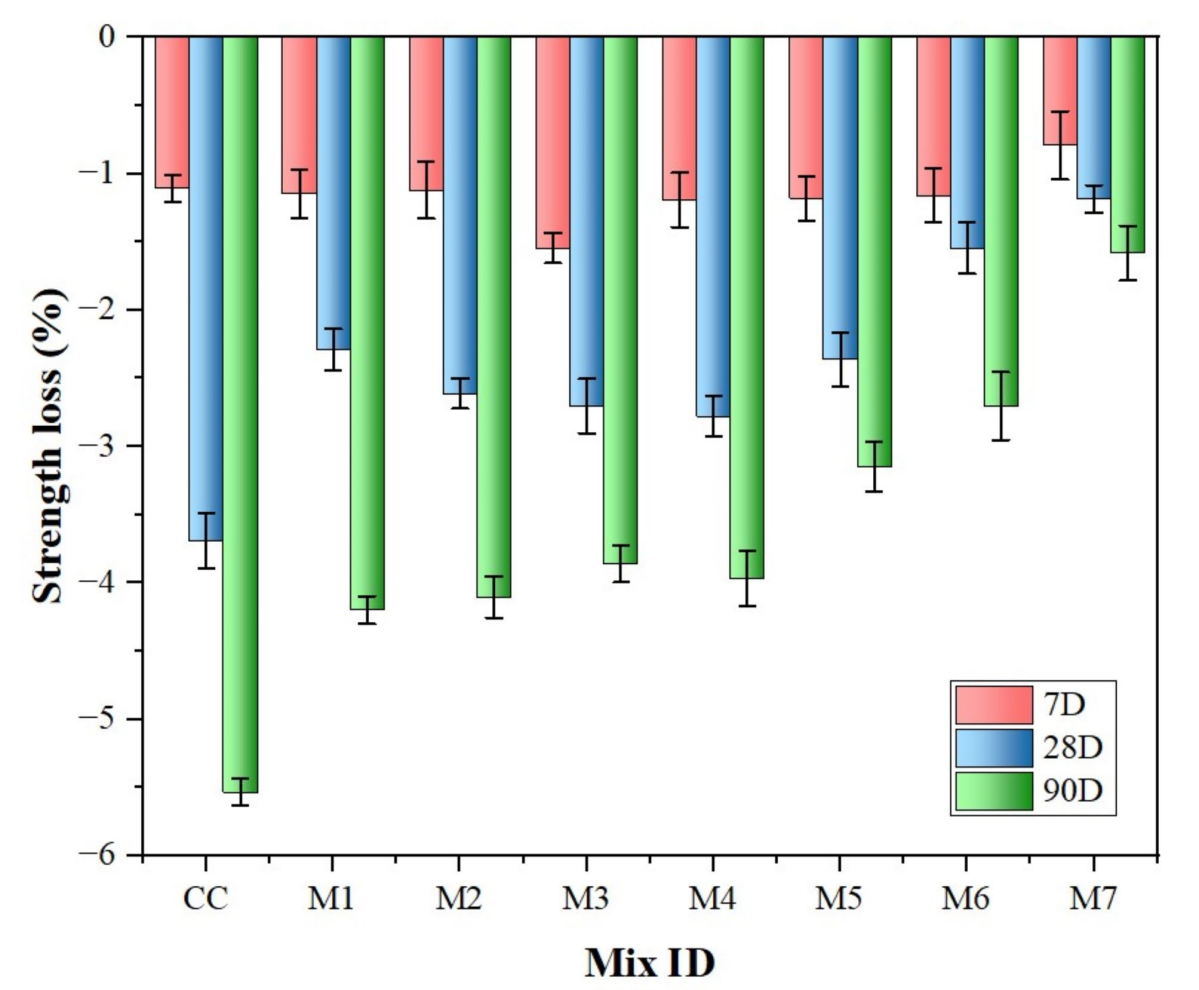
Effect of weight loss after sulphate attack after 7th, 28th and 90th days.

Sulphate attack on concrete is a multifaceted process that involves both physical salt attacks from salt crystallization and chemical reactions with sulphate ions from the surrounding environment. The formation of ettringite, a crystalline substance, occurs when sulphates react with aluminates in CC. This reaction leads to cracking, loss of strength, and disintegration of concrete owing to tensile stresses. Visual inspections were conducted to assess the signs of damage, such as cracks or spalling, after 7th ,28th and 90th days of curing in sulphate solution. All specimens were severely affected by spalling at the edges, with white traces deposited on their surfaces. Nonetheless, the specimen containing MK 12.50% and NS 2% showed minimal visible deterioration and weight loss. The formation of ettringite was anticipated due to the reaction between sulphates and the aluminates in MK, yet the pore refinement attributed to the MK and NS particles reduced the penetration of sulphate ions. As shown in Fig. 15, MK-NS blended concrete specimens experienced only 1.58% strength loss compared to normal concrete, which experienced 5.54% loss. This shows that concrete modified with MK and NS is more resistant to sulphate attack, demonstrating the significance of the MK-NS combination in enhancing durability against chemical aggressions.

### Comparison with previous research studies

In the current study, increasing the content of NS in concrete resulted in significant improvements in both water absorption and chloride permeability compared to previous research. Shafiq et al.^62^ found that concrete containing MK showed a 57% reduction in water penetration depth compared to the control mix, with 47% less penetration in concrete made of 5% MK with 1% NS. Similarly, in this study, the most significant reduction in water absorption occurred with 20% MK, with further enhancements observed when MK and NS were combined. Bhat et al.^63^reported that water absorption in mixes containing NS and MK ranged from 3.50 to 4.80%, which aligns with the findings of the current study. Furthermore, the rapid chloride permeability (RCP) values in this study follow a similar trend to earlier research, with mixes containing NS showing moderate to high permeability, while MK mixes demonstrated low to moderate RCP values, indicating improved chloride ion binding and reduced permeability. The optimized mix of 12.50% MK and 2% NS exhibited very low RCP values, showing superior resistance to chloride penetration compared to earlier studies. Additionally, the combination of MK and NS in concrete also enhanced resistance to acid and sulphate attacks. Moreover, the combined mix of 12.50% MK and 2% NS in this study delivers better durability than the mixes in various previous research, thereby exhibiting significantly enhanced overall performance.

### Microstructural analysis

#### XRD

**Fig. 16:**
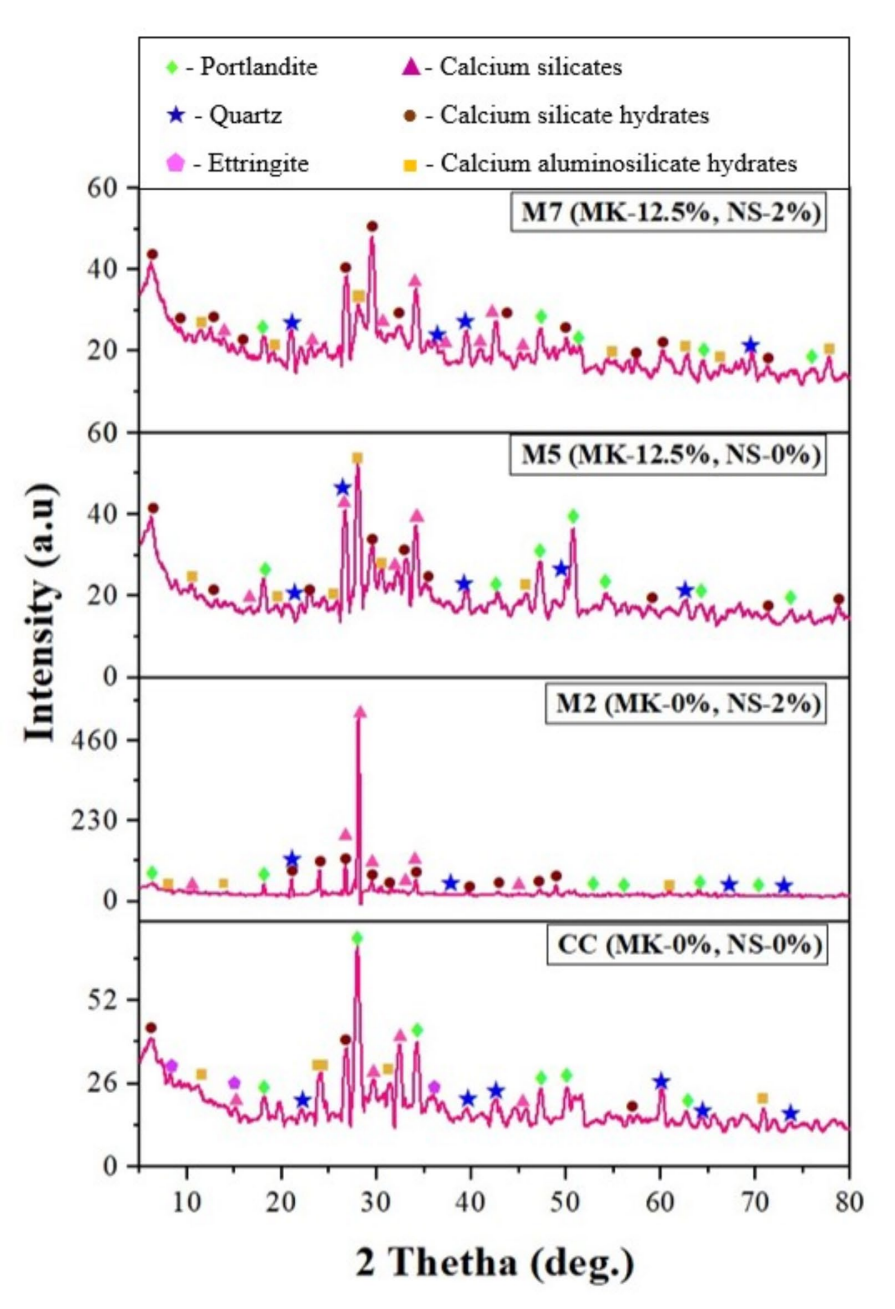
XRD patterns of CC, MK and NS modified concrete mix at 28th day.

Figure 16 shows the XRD spectra of the concrete mixtures that include the optimum mixes of NS and MK. After 28th day of hydration, the hydration phases of conventional concrete (CC), optimized mixes of NS (M2) and MK (M5) and hybrid NS-MK blended mix (M7) were analysed using an X-Pert high score to match the hydration products, including portlandite (P), Calcium Aluminosilicate Hydrate (CASH), Calcium Silicate (CS), Calcium Silicate Hydrate (CSH), quartz (Q), and ettringite (E).

The XRD spectra of Mix 2 (NS added at a concentration of 2%), as depicted in Fig. 16, revealed a decrease in portlandite precipitation relative to the conventional mix^70^. The XRD graph shows that the high intensity in mix 2 indicates a high silica concentration in the matrix, which contributes to the strength. Similarly, the XRD pattern for mix M5 shows increased quartz peaks and reduced portlandite compared to CC. These results align with previous studies, highlighting intense pozzolanic activity and the formation of secondary hydration products, such as additional C-S-H gel, at a 12.50% MK replacement rate^73^. At the same time, the peaks related to MK became more prominent, indicating an excessive amount of secondary hydration products for MK replacement. Likewise, the peaks associated with MK and its reaction products, including the formation of extra C-S-H gel, became increasingly noticeable, suggesting a strong pozzolanic activity for MK replacement of 12.50%.

Moreover, the XRD patterns of MK-NS modified concrete (M7) showed no significant differences in the phase compositions of the CC and mix 7. However, the portlandite peak’s intensity changed in the XRD pattern, suggesting that the addition of MK and NS boosted the crystalline phase’s consumption in the pozzolanic reaction. Remarkably, these results concur with several other research findings, supporting the idea that improved hydration caused by the transformation of portlandite into secondary C-S-H gel, leading to improved strength of all mixes^74,75^. This was apparent from the decrease in the magnitude of the peaks related to portlandite in the XRD pattern of the blended concrete. Likewise, the XRD pattern of the mix 7 showed a decline in the intensity of the ettringite phase in comparison to that of the CC. Furthermore, the reduced pore volume resulting from the smaller crystal structures of the C-A-S-H products in the mix 7 significantly improves the concrete’s strength. Moreover, the XRD pattern showed no new phases, indicating that the structure of the hydration products in the amended concrete remained constant, resulting in increased mechanical strength.

#### FE-SEM

**Fig. 17:**
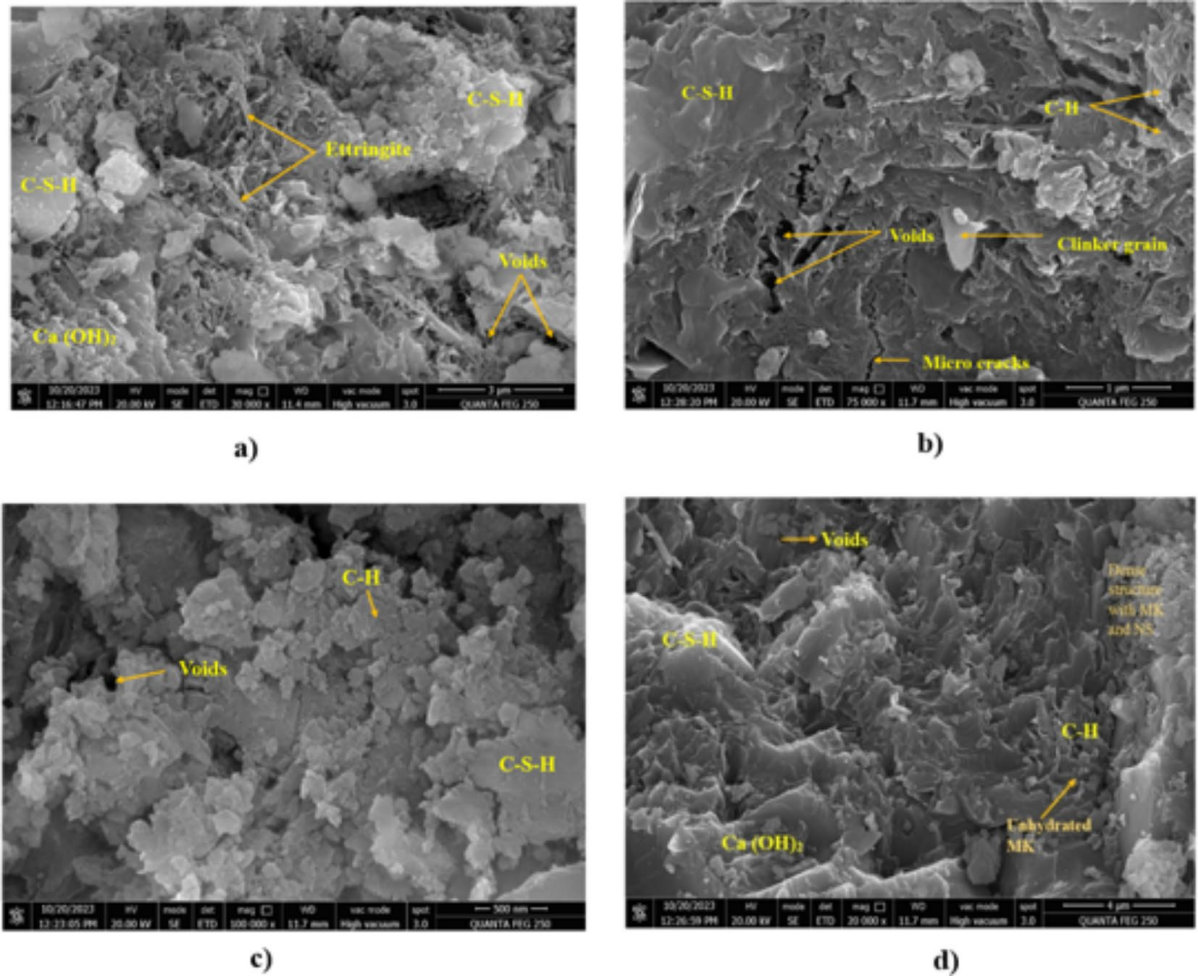
FE-SEM micrographs at 28th day of curing of (**a**) CC (**b**) MK Concrete (**c**) NS Concrete (**d**) MK-NS blended concrete.

An electron microscope reveals the complex ITZ between aggregates and cement mortar, characterised by C-S-H gel, portlandite crystals, voids, and ettringite. As shown in Fig. 17, FE-SEM analyses of conventional concrete (CC), NS-modified concrete (M2), MK-modified concrete (M5), and MK-NS-blended concrete (M7) revealed distinct microstructures.

In CC, cement particles appear as clinker crystals of various sizes in porous microstructures with irregular voids. Furthermore, MK-blended concrete exhibits increased density as an extra C-S-H gel is formed when MK reacts with calcium hydroxide, whereas due to the incorporation of NS particles in concrete, NS blended concrete mixes have a denser microstructure, as depicted in Fig. 17b, c.

Consequently, owing to the synergistic effects of MK and NS in MK-NS blended concrete, the microstructure becomes even denser, as evidenced by the FE-SEM images, Fig. 17 (d) showing various hydration products, notably a rise in C-S-H gel formation. Based on previous research, adding NS and MK reduces micropores and enhances compactness^62,63^. These improvements are similar to the results of Ghafari et al. for optimising the compactness of the concrete matrixes by adding NS^76^. In addition, Zhaohui et al. discovered that embedding MK particles in transition zones improved microstructure compactness, resulting in increased strength and reduced permeability^77^. Hence, the concrete matrix exhibiting the combination of 12.50% MK and 2% NS improved microstructural properties and provided greater mechanical strength and durability than the other mixes.

### Environmental impact assessment

#### Embodied carbon emission

Embodied carbon in concrete refers to the CO_2_ emissions generated throughout its life cycle from raw materials to disposal. Table 5 quantifies the CO_2_ emissions per kilogram of material over the whole lifecycle, encompassing the production process, transportation, and mixing. To calculate embodied carbon quantities for concrete mixes, multiply the weight of each material used to make one cubic meter of concrete by the respective emission factor and sum these values.

**Table 5:** Total CO_2_ emissions emitted to produce 1 m^3^ MK-NS concrete.

Materials	KgCO_2_/kg	CO_2_ emissions for 1m^3^ of concrete (kgCO_2_/m^3^)								Ref
		CC	M1	M2	M3	M4	M5	M6	M7	
OPC	0.82	302.88	302.88	302.88	302.88	287.74	265.02	242.31	265.02	^81^
MK	0.33	–	–	–	–	6.09	15.23	24.38	15.23	^82^
NS	8.4e^-4^	–	0	3.10e^-3^	6.20e^-3^	9.30e^-3^	–	–	3.10e^-3^	^83^
FA	1.39e^-2^	9.62	9.62	9.62	9.62	9.62	9.62	9.62	9.62	^4^
CA	4.08e^-2^	52.81	52.81	52.81	52.81	52.81	52.81	52.81	52.81	^4^
Water	1.96e^-4^	0.03	0.03	0.03	0.03	0.03	0.03	0.03	0.03	^84^
SP	0.72	0.53	0.80	1.06	1.33	0.50	0.70	0.85	1.40	^85^
**Total**		**365.87**	**366.14**	**366.40**	**366.67**	**356.35**	**340.41**	**330**	**341.11**	

The total CO_2_ emissions from concrete are predominantly driven by OPC, as detailed in Table 5. For the CC mix, most CO_2_ emissions are from OPC. In contrast, the CO_2_ contribution of OPC has been significantly reduced by replacing 20% of it with MK (M6 mix), which reduced it from 365.87 to 330 kgCO_2_/m^3^. Although, after the 28th day, the strength of the CC was estimated to be 36.08 MPa, despite the strength of M6 concrete was found to be 33.64 MPa, which means it leads to a 6% reduction in strength. Additionally, NS was used as an additive material in CC, and its replacement percentage showed a slight variation in CO_2_ emissions (M1-M3 mixes) compared with CC. However, adding NS to concrete alongside MK increases its strength while reducing embodied carbon emissions. As a result of using MK-NS blended concrete, the CO_2_ contribution decreased from 365.87 kgCO_2_/m^3^ to 341.11 kgCO_2_/m^3^ when compared with the CC mix.

Thus, the overall embodied CO_2_ emissions can be reduced by replacing SCMs with OPC. Nevertheless, this study found that a higher substitution level of OPC for SCMs leads to a lower strength. Consequently, reducing CO_2_ emissions is essential, but maintaining strength is equally important, thus making micro-nano blends in concrete vital. This leads to an understanding that micromaterials can reduce CO_2_ emissions while achieving high strength with low nanomaterial concentrations, resulting in economical concrete with lower environmental emissions and high strength.

### Eco-strength efficiency

The eco-strength efficiency of concrete assesses its environmental sustainability by considering the ratio of its mechanical strength to its embodied carbon footprint (Eq. (17)). This study aimed to evaluate the efficiency of mechanical performance while reducing its environmental impact.

17 $${\text{Eco}}-{\text{strength efficiency}} =\frac{28\text{t}\text{h}\:\:\text{d}\text{a}\text{y}\:\text{c}\text{o}\text{m}\text{p}\text{r}\text{e}\text{s}\text{s}\text{i}\text{v}\text{e}\:\text{s}\text{t}\text{r}\text{e}\text{n}\text{g}\text{t}\text{h}\:}{\text{t}\text{o}\text{t}\text{a}\text{l}\:\text{e}\text{m}\text{b}\text{o}\text{d}\text{i}\text{e}\text{d}\:\text{c}\text{a}\text{r}\text{b}\text{o}\text{n}\:\text{o}\text{f}\:\:\text{t}\text{h}\text{e}\:\text{c}\text{o}\text{n}\text{c}\text{r}\text{e}\text{t}\text{e}\:}$$

**Fig. 18:**
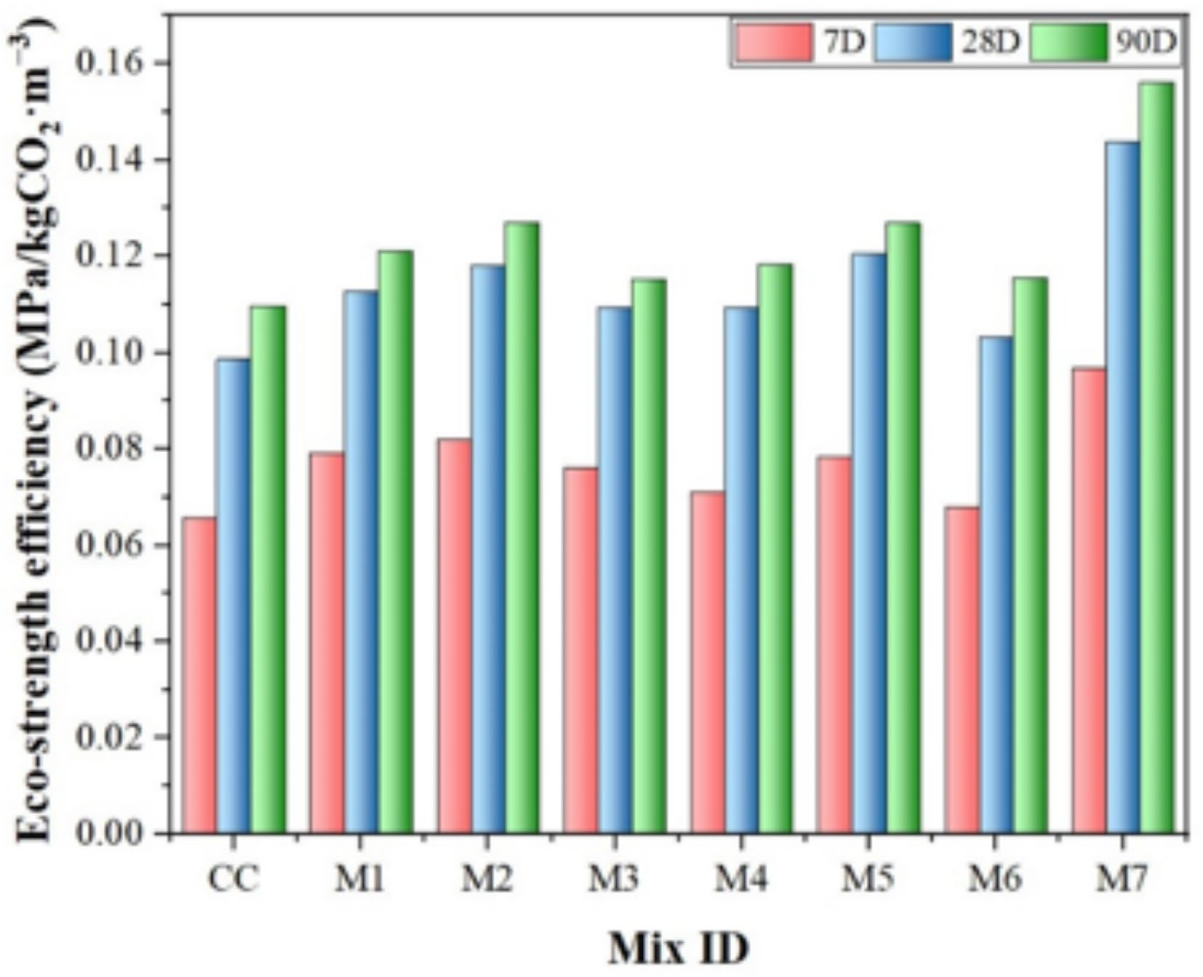
Eco-strength efficiency of different mixes of MK and NS-modified concrete.

The concrete’s eco-strength efficiency is impacted by its durability and carbon emissions, with higher ecological efficiency indicating a more sustainable mix. Figure 18 demonstrates that the CC did not involve any cement substitution and had the lowest eco-strength efficiency. Moreover, the efficacy of eco-strength increases as the level of OPC replacement increases. The eco-strength performance of the CM mix was measured to be 0.066 MPa/kgCO_2_·m^− 3^ on the 7th day. It then increased progressively to 0.099 MPa/kgCO_2_·m^− 3^ on the 28th day and further to 0.11 MPa/kgCO_2_·m^− 3^ on the 90th day. The rate of increase decreases as the concrete ages. Regarding mix 1, mix 2, and mix 3, there was a significant disparity in efficiency between the 7th and 28th days, with a range of 2.50–5%. The variation could be ascribed to the inclusion of NS. However, for mix 4, mix 5, and mix 6, the variation in efficiency between the 7th and 28th days was limited to a range of 0.50–2.50%. The delayed strength increase observed at an early age may be attributed to the pozzolanic reaction induced by MK. Furthermore, it is evident that all mixtures, with the exception of CC, demonstrate a notable enhancement in efficiency. This can be ascribed to the enhanced durability resulting from the pozzolanic reaction. Moreover, the lowest carbon dioxide emission was found for MK replacement at 20%, but the eco-strength efficiency was decreased. Replacement of cement with 12.50% MK and 2% NS addition on 28th day is 32% more efficient than the efficiency of CC. MK-NS blended concrete can improve the microstructure of concrete, thereby increasing its strength and overall durability, but it also exemplifies a sustainable solution for eco-efficient construction practices.

## Conclusion

The study investigated the behaviour of blended concrete using micro (MK) and nano (NS) materials to enhance the strength and durability of hardened concrete by refining its microstructure. The findings highlight the possible use of MK-NS blends in concrete to promote sustainable construction practices by decreasing carbon emissions. The integration of MK with NS enhances the mechanical properties of concrete, resulting in improved compressive strength and overall structural integrity. Likewise, these mixes provide improved resistance to chloride penetration, acids, and sulfate attacks. The microstructure is significantly refined, as demonstrated by XRD and FE-SEM studies, revealing a denser, more compact matrix with less porosity. This enhancement in microstructure enhances resilience to environmental degradation. Consequently, MK-NS blended concrete is suitable for use in challenging situations, including bridges, composite structures, highways, marine infrastructure, and high-rise buildings, where durability, superior strength, and resilience to environmental factors are essential. This blend’s capacity to diminish environmental effect while preserving or enhancing structural performance establishes it as a sustainable solution for future infrastructure development. Furthermore, this study reveals the following findings from the inclusion of MK and NS in concrete mixtures:

- A concrete mix containing 12.50% MK and 2% NS demonstrates 30 − 40% increase in strength when compared to CC owing to the transformation of portlandite into secondary CSH and the enhancement of pore structure and hydration.
- The blended concrete exhibited reduced porosity due to the combination of MK and NS, as evidenced by improved resistance to water absorption, sorptivity and chloride penetration. NS enhanced the pore structure, while MK bonded chloride ions with aluminates. The 12.50% MK and 2% NS blends demonstrated superior durability.
- The conventional concrete exhibited the lowest resistance to acid and sulphate attacks, whereas MK and NS blends showed better resistance on 90th day.
- The analysis revealed that the concrete with 12.50% MK and 2% NS had superior properties compared to other mixtures. XRD patterns indicated improved hydration and pore structure, leading to better mechanical properties. FE-SEM micrographs showed compact nanostructures with effective NS dispersion and a strong MK interface.
- Replacing 20% OPC with MK reduced CO_2_ emissions from 365.87 to 330 kgCO_2_/m^3^, despite a 6% decrease in strength. NS, combined with MK, lowered CO_2_ emissions to 341.11 kgCO_2_/m^3^, demonstrating that micro-nano blends can achieve high strength with a reduced environmental impact.

A 2% addition of NS and replacing 12.50% of MK with OPC improved eco-strength efficiency by 32% at 28th days compared to the CC, illustrating the environmental as well as structural benefits of SCMs.

## Data Availability

“The datasets used and/or analysed during the current study available from the corresponding author on reasonable request”.

## Abbreviations

Al_2_O_3_: Aluminium oxide
CaO: Calcium oxide
C-A-S-H: Calcium aluminosilicate hydrate
CO_2 −_: Carbon dioxide
C-S-H: Calcium silicate hydrate
FE-SEM: Field-emission scanning electron microscopy
Fe_2_O_3_: Ferric oxide
K_2_O: Potassium oxide
MgO: Magnesium oxide
Na_2_O: Sodium oxide
SiO_2_: Silicon dioxide
SO_3_: Sulphur trioxide
TiO_2_: Titanium dioxide
